# Supplementary material for: Dietary fibre and whole grains in diabetes management: Systematic review and meta-analyses
Source: PLoS Med. 2020 Mar 6;17(3):e1003053. doi: 10.1371/journal.pmed.1003053 (PMC7059907; doi:10.1371/journal.pmed.1003053)
Supplement: S1 Appendix — PICO, Population, Intervention, Control, and Outcomes. (DOCX) [file pmed.1003053.s001.docx]

**S1 Appendix. PICO tables: Fibre and whole grains in diabetes management**

**S1 Text.** PICO tables for these questions were developed by a reference group of the DNSG in 2018.

**Questions**

1. What is the effect of consuming higher fibre diets on cardiometabolic risk factors in those with prediabetes, gestational diabetes, type 1 diabetes, and type 2 diabetes.

**Populations**

Adults diagnosed with prediabetes, gestational diabetes, type 1, or type 2 diabetes as defined by papers.

**A priori subgroup analyses or meta regression where data are available:**

| Diabetes type | Type 1, type 2, prediabetes, and gestational diabetes |
| --- | --- |
| Participant characteristics | Weight: normal, overweight, obese  Purposeful recruitment by age group  Sex: M, F  SES: low, middle, high  Medication: taking insulin, not taking insulin |
| Study location | Country income: low, middle, high  Ethnicity: as described |
| Study quality | Cochrane tool for assessing risk of bias, and parameters of imputing values to enable inclusion into the meta analysis. |

**Interventions**

The provision of whole grains or foods containing fibre or advice to consume whole grains or foods containing fibre for a minimum six weeks.

**A priori subgroup analyses or meta regression where data are available:**

| Trial parameters | Duration: 6 weeks, 8 weeks, >3 months, 12 months  Treatment type: lifestyle, oral medication, insulin |
| --- | --- |
| Intervention parameters | Naturally occurring fibres or extracted or synthetic  ‘minimally processed’ whole grains or unspecified  Type: dietary advice or food provision  Amount: volume of fibre or whole grains (dose response if data are available)  Fibre type: all fibre or just one type (i.e inulin) viscosity, solubility.  Wholegrain type: all grains or just one type (i.e oats) |

**Comparison**

A high wholegrain or fibre diet compared with a control diet without advice to reduce energy intake.

**A priori subgroup analyses or meta regression where data are available:**

| Control diet | Advice to eat normal diet, advice to change amount of fibres or whole grains, no advice |
| --- | --- |
| Trial design | Parallel, crossover |
| Unintended weight loss | no weight change, significant difference in weight between groups |

**Outcomes**

| Change in glycated haemoglobin | Primary |
| --- | --- |
| Change in intermediate cardiovascular risk factors and body weight | Secondary |
| Change in glycated albumin | Secondary |
| Change in fasted or mean blood glucose concentration | Secondary |
| Change in area under the curve for oral glucose tolerance or meal tests | Secondary |
| Change in reported measures of glycaemic variability | Secondary |
| Change in markers of insulin concentration | Secondary |

Note: the search strategy will also identify prospective observational studies that report on cohorts diagnosed with prediabetes, gestational diabetes, type 1 diabetes, or type 2 diabetes. For these studies, the exposure quantiles of wholegrain or fibre intake and RR or HR of all cause or CVD mortality will be used. Subgroup analysis for prospective observational studies (where identified) will be by diabetes type, by marker of carbohydrate quality, and by study quality as measured by the Newcastle Ottawa Scale.
