# Supplementary material for: Dietary fibre and whole grains in diabetes management: Systematic review and meta-analyses
Source: PLoS Med. 2020 Mar 6;17(3):e1003053. doi: 10.1371/journal.pmed.1003053 (PMC7059907; doi:10.1371/journal.pmed.1003053)
Supplement: S2 Appendix — Table A: Description of identified prospective studies. Table B: Description of identified intervention trials. Fig A: Cochrane risk of bias tool summary for the trials identified as eligible for this review. (DOCX) [file pmed.1003053.s002.docx]

**S2 Appendix. Identified studies**

**Online search terms**

Search #1

diabet* OR prediabet* or T1* OR T2* OR NIDDM OR type 1 diabetes OR type 2 diabetes OR IDDM OR non-insulin dependent OR non insulin dependent OR insulin dependent OR adult onset diabetes OR adult-onset diabetes OR juvenile-onset diabetes OR juvenile onset diabetes

Search #2

blood glucose control OR glycat* OR hba1c OR a1c OR fasted blood glucose OR mean blood glucose OR OGTT OR oral glucose tolerance test OR glycaemi* OR glycemi* OR blood pressure OR systolic OR diastolic OR SBP OR DBP OR blood lipid profile OR cholesterol OR LDL OR HDL OR triglycerides OR insulin OR HOMA IR OR CRP OR C-reactive OR C reactive OR waist circumference OR WC OR BMI OR body mass index OR body weight

Search #3

Dietary carbohydrate OR whole grain OR wholegrain* OR whole-grain OR dietary fibre OR dietary fiber OR CHO

Search #4

randomised controlled trial OR randomized controlled trial OR parallel trial OR crossover trial OR clinical trial OR controlled trial OR systematic review OR prospective observational study OR cohort study OR nested case-control OR nested case control OR nested cohort

Combine searches

#1 AND #2 AND #3 AND #4

**S2 Table A: Description of identified prospective studies**

| ID | Cohort | Participants | Exposure | Outcomes | Adjustments | Newcastle Ottawa Score |
| --- | --- | --- | --- | --- | --- | --- |
| **Prospective cohort of adults with type 2 diabetes** | | | | | | |
| Burger 2012 **Europe ^1^** | Subset of the EPIC study | 6192 adults aged 35-70 with T2 diabetes followed for a mean 9.2 years | Dietary fibre intake obtained from FFQ or Diet history | 791 deaths, 306 due to cardiovascular disease | Age, sex, country, smoking, smoking duration, education, BMI, WHR, physical activity, alcohol intake, menopausal status, and HRT use, diabetes duration, insulin use, and glycated haemoglobin level. | Exposed cohort representative: *  Non exposed cohort representative: *  Exposure ascertainment: 0  Outcome not present at start: *  Controlled for weight: *  Controlled for age: *  Outcome assessment: *  Follow up duration: *  Follow up adequacy: * |
| **Prospective cohort of adults with type 1 diabetes** | | | | | | |
| Schoenaker 2012 **Europe ^2^** | EURODIAB | 2108 people aged 15-60 with T1 diabetes followed for a mean 7.3 years | Dietary fibre intake obtained from three day diet record | 46 deaths, 15 due to cardiovascular disease | Age, sex, energy intake, diabetes duration, HbA1c, smoking status, physical activity, alcohol intake, saturated fat intake. | Exposed cohort representative: *  Non exposed cohort representative: *  Exposure ascertainment: *  Outcome not present at start: *  Controlled for weight: 0  Controlled for age: *  Outcome assessment: *  Follow up duration: *  Follow up adequacy: * |

*****Authors from both prospective studies provided additional analyses and information to enable data use within the current study.

1. Burger KN, Beulens JW, van der Schouw YT, et al. Dietary fiber, carbohydrate quality and quantity, and mortality risk of individuals with diabetes mellitus. PLoS One 2012; 7(8): e43127.

2. Schoenaker DAM, Toeller M, Chaturvedi N, Fuller JH, Soedamah-Muthu SS, Group EPCS. Dietary saturated fat and fibre and risk of cardiovascular disease and all-cause mortality among type 1 diabetic patients: the EURODIAB Prospective Complications Study. Diabetologia 2012; 55(8): 2132-41.

**S2 Table B: Description of identified intervention trials**

| ID | Design | Participants | Daily intervention (I) | Daily control (C) | Outcomes | Duration | Fibre difference between I & C | Cochrane Risk of Bias tool |
| --- | --- | --- | --- | --- | --- | --- | --- | --- |
| **Trials of adults with type 1 diabetes** | | | | | | | | |
| Giacco 2000 **Italy ^3^** | Randomised Parallel Trial | 54 with T1 diabetes. 25 (16F) in control: age 26.2y, BMI 24.2. HbA1c 8.8% insulin dose 42.7 U/day. 29 (17F) in intervention: age 29.5y. BMI 23.6. HbA1c 8.8% and insulin dose 47.9 U/day | Advice to consume a high fibre diet | Diet with limited consumption of high fibre foods | HbA1c, mean glucose, body weight, total cholesterol, HDL, and triglycerides | 24 weeks | 21.3g | Sequence generation: Unclear  Allocation concealment: Unclear  Blinding of participants: Unclear  Blinding of outcome: Unclear  Incomplete data: Low  Selective reporting: Low  Other: Low |
| Venhaus 1988 **Germany ^4^** | Randomised Crossover Trial | 10 with T1 diabetes (2F). 27y. 22.6 BMI. HbA1c 6.4%. Duration 13y | Advice to consume diet high in unrefined carbohydrates diet | Advice to avoid wholegrain foods  and limit intake of fruits and vegetables | HbA1c, body weight, total cholesterol, HDL, and triglycerides | 6 weeks | 17.6g | Sequence generation: Unclear  Allocation concealment: Unclear  Blinding of participants: Unclear  Blinding of outcome: Unclear  Incomplete data: Low  Selective reporting: Unclear  Other: Low |
| **Trials of adults with pre diabetes** | | | | | | | | |
| Canfora 2017 **Netherlands ^5^** | Randomised Parallel Trial | 44 with prediabetes. 23 (11F) control: 58.4y. BMI 32.3. Hba1c 5.6% 21 (10F) intervention: 59.2y. BMI 33.3. HbA1c 5.6% | 5g galacto-oligosaccharides in low fat yoghurt drink at each main meal | Placebo powder in low fat yoghurt drink with each meal | Fasting glucose, fasting insulin, body weight, BMI, and triglycerides | 12 weeks | 15g | Sequence generation: Unclear  Allocation concealment: Unclear  Blinding of participants: Unclear  Blinding of outcome: Unclear  Incomplete data: Low  Selective reporting: Low  Other: Low |
| Daimon 2011 **Japan ^6^**  Not available in English | Randomised Parallel Trial | 61 with prediabetes. 31 (19F) control, 30 (15F) intervention. 59.3y. BMI 24.9 | 1g beta-glucan before or after evening meal | Roast barley flour before or after evening meal | Fasting glucose and fasting insulin | 12 weeks | 1g | Sequence generation: Unclear  Allocation concealment: Unclear  Blinding of participants: Low  Blinding of outcome: Low  Incomplete data: Low  Selective reporting: Unclear  Other: Low |
| Errazuriz 2017 **USA ^7^** | Randomised Parallel Trial | 28 with prediabetes. 13 (3F) control: 60y. BMI 32. HbA1c 5.5%. 15 (8F) intervention: 63y. BMI 31. HbA1c 5.6% | Advice to follow high fibre diet using beans and fibre bars | Advice to follow habitual diet | Fasting insulin and insulin sensitivity | 12 weeks | 19.6g | Sequence generation: Unclear  Allocation concealment: Unclear  Blinding of participants: Unclear  Blinding of outcome: Unclear  Incomplete data: Low  Selective reporting: Low  Other: Low |
| Garcia 2007 **Germany ^8^** | Randomised Crossover Trial | 22 (7F) with prediabetes. 55.5y. BMI 30.1. HbA1c 5.6% | 2 bread rolls and powder containing Arabinoxylan | 2 bread rolls and placebo powder | Fasting glucose, basting insulin, body weight, and triglycerides | 6 weeks | 12.4g | Sequence generation: Unclear  Allocation concealment: Unclear  Blinding of participants: Unclear  Blinding of outcome: Unclear  Incomplete data: Unclear  Selective reporting: Low  Other: Low |
| Kobayakawa 2013 **Japan ^9^** | Randomised Parallel Trial | 30 with prediabetes. 15 in each arm, 53.5y | 2.5g oat bran, inulin, guar gum mix, konjac and seaweed extract (all fibre) at each main meal | 2.5g starch without fibre with each meal | HbA1c, fasting glucose, fasting insulin, HOMA IR, body weight, BMI, and waist circumference | 12 weeks | 7.5g | Sequence generation: Low  Allocation concealment: Low  Blinding of participants: Unclear  Blinding of outcome: Unclear  Incomplete data: Low  Selective reporting: Low  Other: Low |
| Lankinen 2011 **Finland ^10^** | Randomised Parallel Trial | 69 (34F) with prediabetes. Control: 59y. BMI 31.0. Intervention: 58y. BMI 31.4 | Advice to follow wheat germ enriched diet and have breads with low postprandial glucose and insulin response | Advice to follow habitual diet and have refined wheat cereal products. Restricted wholegrain intake | Fasting glucose, postprandial glucose, fasting insulin, HOMA IR, and postprandial insulin | 12 weeks | 12.2g | Sequence generation: Unclear  Allocation concealment: Unclear  Blinding of participants: Unclear  Blinding of outcome: Unclear  Incomplete data: Low  Selective reporting: Low  Other: Low |
| Peterson 2018 **USA ^11^** | Randomised Parallel Trial | 59 (39F) with prediabetes. Control: 55y. BMI 35.7.  Intervention: 54y. BMI 35.5. | 45g RS2 high-amylose maize | Isocaloric amount of Amioca cornstarch | HbA1c, fasting glucose, fasting insulin, total cholesterol, LDL, HDL, triglycerides, SBP, DBP, and CRP | 12 weeks | 45g | Sequence generation: Unclear  Allocation concealment: Unclear  Blinding of participants: Unclear  Blinding of outcome: Unclear  Incomplete data: Low  Selective reporting: Low  Other: Low |
| Trials of adults with pre diabetes or type 2 diabetes | | | | | | | | |
| Kang 2014 **Korea ^12^** | Randomised Parallel Trial | 185 (146F) with prediabetes or T2. 50.3y. BMI 25.3 | Advice to replace white rice with one third legumes, one third barleys, and one third whole grains with each meal | Advice to follow habitual diet (white rice) | Fasting glucose, fasting insulin, HOMA IR, HDL, and triglycerides | 12 weeks | 3.4g or 1.3g by subgroup | Sequence generation: Unclear  Allocation concealment: Unclear  Blinding of participants: Unclear  Blinding of outcome: Unclear  Incomplete data: Unclear  Selective reporting: Low  Other: Low |
| Kim 2014 **Korea ^13^** | Randomised Parallel Trial | 99 (32F) with prediabetes or T2. Control 55.4y. BMI 24.1. Intervention 56.3y. BMI 24.0 | Advice to replace white rice with black soy beans, barley and brown rice mix | Advice to follow habitual diet (white rice) | HbA1c, fasting glucose, postprandial glucose, fasting insulin, HOMA IR, postprandial insulin, BMI, blood pressure, c-reactive protein, total cholesterol, HDL, LDL, and triglycerides | 12 weeks | 4.9g | Sequence generation: Low  Allocation concealment: Unclear  Blinding of participants: Unclear  Blinding of outcome: Unclear  Incomplete data: Unclear  Selective reporting: Low  Other: Low |
| Schwab 2006 **Finland ^14^** | Randomised Parallel Trial with two relevant intervention arms | 66 with prediabetes or T2. 22 (12F) in control: BMI 28.8. 22 (12F) in PDX intervention: 55y. BMI 28.4. 22(13F) in SBP intervention 51y BMI 29.4 | 8g sugar beet pectin or polydextrose drink at two meals | No drink given | HbA1c, fasting glucose, body weight, BMI, blood pressure, total cholesterol, LDL, HDL, and triglycerides | 12 weeks | 12.9g or 15.2g by subgroup | Sequence generation: Unclear  Allocation concealment: Unclear  Blinding of participants: Low  Blinding of outcome: Unclear  Incomplete data: Unclear  Selective reporting: Low  Other: Low |
| Trials of adults with type 2 diabetes | | | | | | | | |
| *Abutair 2016 **Gaza ^15^** | Randomised Parallel Trial | 36 (20F) with T2. BMI 31.7. HbA1c 8.5%. Included oral medication | 7g psyllium before lunch and 3.5 g psyllium before dinner | Advice to follow habitual diet | HbA1c, fasting glucose, body weight, BMI, blood pressure, total cholesterol, LDL, HDL, and triglycerides | 8 weeks | 10.5g | Sequence generation: Unclear  Allocation concealment: Unclear  Blinding of participants: Unclear  Blinding of outcome: Unclear  Incomplete data: Low  Selective reporting: Low  Other: Low |
| Aliasgharzadeh 2015 **Iran ^16^** | Randomised Parallel Trial | 55F with T2. 25 in control. 49.2y. BMI 31.8. Duration 6.9y. Hba1c 8.2%. 30 in intervention. 49.6y. BMI 30.8. Duration 5.2y. HbA1c 7.8% | 5g resistant dextrin at breakfast and dinner | Placebo powder at breakfast and dinner | HbA1c, fasting glucose, fasting insulin, insulin sensitivity, HOMA IR, body weight, BMI, and c-reactive protein | 8 weeks | 10g | Sequence generation: Low  Allocation concealment: Low  Blinding of participants: Low  Blinding of outcome: Unclear  Incomplete data: Low  Selective reporting: Low  Other: Low |
| Aro 1981 **Finland ^17^** | Randomised Crossover Trial | 9 (4F) with T2. 53y. Duration 6.5y. treated with diet or oral drugs | 7g guar gum with each main meal | Placebo powder with each main meal | Fasting glucose | 3 months | 16.8g | Sequence generation: Unclear  Allocation concealment: Unclear  Blinding of participants: Unclear  Blinding of outcome: Unclear  Incomplete data: Low  Selective reporting: Low  Other: Low |
| Babiker 2018 **Sudan ^18,19^** | Randomised Parallel Trial | 91(73F) with T2. 50y. Duration 5y. HbA1c 9.4%. Treated with oral drugs. | 30g gum arabic in the morning | 5g pectin placebo in the morning | HbA1c, fasting glucose, body weight, BMI, waist circumference, SBP, DBP, total cholesterol, LDL, HDL, and triglycerides | 3 months | 30g | Sequence generation: Low  Allocation concealment: Low  Blinding of participants: Unclear Blinding of outcome: Low  Incomplete data: Low  Selective reporting: Unclear  Other: Low |
| Baker 1988  **UK ^20^** | Randomised Parallel Trial | 30 (20F) with T2. 15(9F) in control: 64.3y. 68.8kg. HbA1c 11.9%. 15(11F) in intervention: 59.1y. 67.2kg. HbA1c 12.0%. Diet and oral medication. | 5g guar gum with each main meal | Placebo powder | HbA1c, fasting glucose | 9 weeks | 15g | Sequence generation: Unclear  Allocation concealment: High  Blinding of participants: Unclear Blinding of outcome: Unclear  Incomplete data: Low  Selective reporting: Unclear  Other: Unclear |
| Calvo-Rubio 1989 **Spain ^21^** | Randomised Parallel Trial | 34 (12F) with T2. 19 (7F) in control: 67y. Duration 5.5y. HbA1c 9.26%.  15 (5F) in intervention 63y. Duration 4y. HbA1c 8.31%. Separate analysis for diet, oral medication, and insulin controlled | 4.75g guar gum with each main meal | Nothing given | HbA1c | 12 weeks | 11.4g | Sequence generation: Unclear  Allocation concealment: High  Blinding of participants: Unclear  Blinding of outcome: Unclear  Incomplete data: Unclear  Selective reporting: Low  Other: Low |
| Chuang 1992 **Taiwan ^22^** | Randomised Crossover Trial | 16 (9F) with T2. 52.1y. Duration 6.4y. All on maximal doses of oral hyperglycaemic agents | 5g guar gum with each main meal | Placebo powder with each main meal | HbA1c, fasting glucose, fasting insulin, total cholesterol, LDL, HDL, and triglycerides | 8 weeks | 15g | Sequence generation: Unclear  Allocation concealment: Unclear  Blinding of participants: Unclear  Blinding of outcome: Unclear  Incomplete data: Low  Selective reporting: Low  Other: Low |
| Cugnet-Anceau 2010 **France and Sweden ^23^** | Randomised Parallel Trial | 53 with T2. 24 in control: 61.8y. BMI 29.03. HbA1c 7.5%. 29 in intervention 61.9y. BMI 30.48. HbA1c 7.3% | 3.5g beta-glucans in soup | Soup with no beta-glucan | HbA1c, fasting glucose, total cholesterol, LDL, HDL, and triglycerides | 8 weeks | 3.5g | Sequence generation: Unclear  Allocation concealment: Low  Blinding of participants: Low  Blinding of outcome: Low  Incomplete data: Low  Selective reporting: Low  Other: Low |
| Dall’Alba 2013 **Brazil ^24^** | Randomised Parallel Trial | 44 (17F) with T2. 21 (8F) in control: Duration 16.9y. BMI 29.3. HbA1c 6.9%. 23 (9F) in intervention: Duration 11.8y. BMI 30.2. HbA1c 6.8%. Insulin and or oral medication | 5g guar gum at lunch and dinner | Nothing given | HbA1c, fasting glucose, body weight, waist circumference, blood pressure, total cholesterol, LDL, and HDL | 6 weeks | 8.6g | Sequence generation: Unclear  Allocation concealment: Unclear  Blinding of participants: Unclear  Blinding of outcome: Unclear  Incomplete data: Unclear  Selective reporting: Unclear  Other: Low |
| **Dehghan 2014 **Iran ^25^**  Not available in English | Randomised Parallel Trial with two relevant intervention arms | 76F with T2. 25 in control: duration 5.3 years. BMI 29.9. 24 in inulin intervention: duration 7.3 years. BMI 31.6. 27 in enriched inulin intervention: duration 8.5 years. BMI 31.9. Diet and oral medication | 5g inulin or oligofructose-enriched inulin at breakfast and dinner | Placebo powder at breakfast and dinner | HbA1c, fasting glucose, fasting insulin, HOMA IR, and c-reactive protein | 8 weeks | 10g | Sequence generation: Low  Allocation concealment: Low  Blinding of participants: Low  Blinding of outcome: Low  Incomplete data: Low  Selective reporting: Unclear  Other: Low |
| Diaz 1990 **Chile ^26^**  Not available in English | Non-Randomised Crossover Trial | 16 (5F) with T2. 52y. Duration 8y. diet or oral medication | 10g sweet lupin hull powder at breakfast for four weeks then 20g for four weeks | Nothing given | HbA1c, fasting glucose, postprandial glucose, total cholesterol, LDL, HDL, and triglycerides | 8 weeks | 6.5g | Sequence generation: High  Allocation concealment: High  Blinding of participants: High  Blinding of outcome: Unclear  Incomplete data: Low  Selective reporting: Low  Other: Low |
| Feinglos 2013 **USA ^27^** | Randomised Parallel Trial with two relevant intervention arms | 37 (12F) with T2. 8 in the control: 56.5y. HbA1c 7.6%. 15 in low dose intervention 61.8y. HbA1c 7.4%. 14 in higher dose intervention 64.8y. HbA1c 7.6%.  diet or oral medication | 3.4g or 6.8g psyllium before breakfast and dinner | Placebo at breakfast and dinner | HbA1c, and fasting glucose | 12 weeks | 3.5g or 7g by subgroup | Sequence generation: Unclear  Allocation concealment: Unclear  Blinding of participants: Unclear  Blinding of outcome: Unclear  Incomplete data: Unclear  Selective reporting: Unclear  Other: Low |
| Feldheim 2013 **Czech Republic ^28^** | Randomised Parallel Trial | 30 with T2. 84y. 24.8 BMI. (Data are presented for 19 diet only participants) | Given bread and crescents enriched with wheat fibre concentrate | Given bread and crescents | HbA1c, fasting glucose, and fasting insulin | 8 weeks | 6g | Sequence generation: Unclear  Allocation concealment: Unclear  Blinding of participants: Unclear  Blinding of outcome: Unclear  Incomplete data: High  Selective reporting: Unclear  Other: High |
| Gargari 2015 **Iran ^29^** | Randomised Parallel Trial | 60 with T2. 32 in control: 49.6y. BMI 30.8. Duration 5.2y. HbA1c 8.2%. 28 in intervention: 49.5y. BMI 31.5. duration 7.5y. HbA1c 7.9%. Oral medication | 5g of resistance starch at breakfast and dinner | Placebo powder at breakfast and dinner | HbA1c, fasting glucose, c-reactive protein, total cholesterol, LDL, HDL, and triglycerides | 8 weeks | 10g | Sequence generation: Low  Allocation concealment: Low  Blinding of participants: Low  Blinding of outcome: Low  Incomplete data: Low  Selective reporting: Low  Other: Low |
| Grunberger 2007 **USA ^30^** | Randomised Parallel Trial | 47(26F) with T2. 27 in control: 58.1y. 20 in intervention 56.8y. Diet, insulin and or oral medication | 6g α-cyclodextrin tablets to have with fat-containing meals | Placebo to have with fat-containing meals | HbA1c, body weight, BMI, and c-reactive protein | 3 months | 6g | Sequence generation: Unclear  Allocation concealment: Unclear  Blinding of participants: Unclear  Blinding of outcome: Unclear  Incomplete data: Unclear  Selective reporting: Low  Other: Low |
| Hagander 1988 **Sweden ^31^** | Randomised Crossover Trial | 14(9F) with T2. 69.1y. BMI 29.2. Duration 3 years. Diet controlled | Given fibre rich foods such as beet-fibre bread | Given low fibre foods such as white bread | HbA1c, fasting glucose, postprandial glucose, fasting insulin, postprandial insulin, total cholesterol, LDL, HDL, and triglycerides | 8 weeks | 35.7g | Sequence generation: Unclear  Allocation concealment: Unclear  Blinding of participants: Unclear  Blinding of outcome: Unclear  Incomplete data: Low  Selective reporting: Low  Other: Low |
| Hagander 1989 **Sweden ^32^** | Randomised Crossover Trial | 12(5F) with T2. 62y. BMI 32.5. HbA1c 7.1%. Oral and diet | Beet fibre added to foods | Low fibre dietary advice | HbA1c, total cholesterol, LDL, HDL, and triglycerides | 8 weeks | 25.6g | Sequence generation: Unclear  Allocation concealment: Unclear  Blinding of participants: Unclear  Blinding of outcome: Unclear  Incomplete data: Unclear  Selective reporting: Low  Other: Low |
| Jenkins 2002 **Canada ^33^** | Randomised Crossover Trial | 23(7F) with T2  Oral and diet | Given wheat bran enriched bread and cereal | Given low fibre bread and cereal | HbA1c, fasting glucose, body weight, blood pressure, c-reactive protein, total cholesterol, LDL, HDL, and triglycerides | 12 weeks | 16.1g | Sequence generation: Unclear  Allocation concealment: Unclear  Blinding of participants: Unclear  Blinding of outcome: Unclear  Incomplete data: High  Selective reporting: Low  Other: Low |
| Karlander 1991 **Sweden ^34^** | Randomised Crossover Trial | 13(4F) with T2. 5 diet controlled: 57.2y. Duration 4.2y. BMI 32.5. 8 on oral medication: 59.6y. Duration 8.0y. BMI 29.8 | Bread with sugar beet fibre for with each meal | Advice to follow habitual diet | HbA1c, fasting glucose, and BMI | 6 weeks | 16g | Sequence generation: Unclear  Allocation concealment: Unclear  Blinding of participants: Unclear  Blinding of outcome: Unclear  Incomplete data: Unclear  Selective reporting: Low  Other: Low |
| Kondo 2017 **Japan ^35^** | Randomised Parallel Trial | 28(10F) with T2. 14 in control: BMI 25.0. HbA1c 6.8%. Duration 14.2y. 14 in intervention: BMI 24.2. HbA1c 6.7%. Oral medication | Given brown rice/barley/ amaranth mix to use in 10 of 21 meals per week | Given white rice to use in 10 of 21 meals per week | HbA1c, fasting glucose, fasting insulin, HOMA IR, body weight, BMI, blood pressure, c-reactive protein, total cholesterol, LDL, HDL, and triglycerides | 8 weeks | 4.2g | Sequence generation: Low  Allocation concealment: Low  Blinding of participants: Low  Blinding of outcome: Low  Incomplete data: Low  Selective reporting: Low  Other: Low |
| Lalor 1990 **UK ^36^** | Randomised Crossover Trial | 19(11F) with T2.  diet controlled but this is a randomised three arm trial where one arm was metformin | 5g guar gum at each main meal | Placebo at each main meal | Fasting glucose, total cholesterol, LDL, HDL, and triglycerides | 12 weeks | 12g | Sequence generation: Unclear  Allocation concealment: Unclear  Blinding of participants: Unclear  Blinding of outcome: Unclear  Incomplete data: Unclear  Selective reporting: Low  Other: Low |
| Li 2016 **China ^37^** | Randomised Parallel Trial | 152(85F) with T2. 80(39F) in control: 59.7y. Weight 72.6kg. HbA1c 8.4%. 79 (46F) in intervention: 59.4y. Weight 74.4kg. HbA1c 8.28%. Diet, insulin and or oral medication | Given 100g wholegrain oats | Given 50g wholegrain oats | HbA1c, fasting glucose, postprandial glucose, body weight, BMI, total cholesterol, LDL, HDL, and triglycerides | 1 year | 4.35g | Sequence generation: Unclear  Allocation concealment: Unclear  Blinding of participants: Unclear  Blinding of outcome: Low  Incomplete data: Low  Selective reporting: Low  Other: Low |
| Moustafa 2007 **Egypt ^38^** | Randomised Parallel Trial | 30(9F) with T2. 15 in control: 52.7y. Duration 13.3y. 15 in intervention: 52.3y. Duration 11.9y.  Treatment is unstated | Given talbina barley porridge | Advice to follow habitual diet | HbA1c, total cholesterol, LDL, and triglycerides | 6 weeks | unknown | Sequence generation: Unclear  Allocation concealment: Unclear  Blinding of participants: Unclear  Blinding of outcome: Unclear  Incomplete data: High  Selective reporting: Unclear  Other: High |
| Pedersen 2016 **UK ^39^** | Randomised Crossover Trial | 29 with T2. 15 in control: 58.1y. Duration 4.0y. BMI 28.2. HbA1c 46.3mmol/mol. 14 in intervention: 567y. Duration 4.6y. BMI 28.0. HbA1c 51.2 mmol/mol. Diet and oral | 5.5g galacto-oligosaccharide powder to mix into food | 5.5g placebo powder to mix into food | HbA1c, fasting glucose, postprandial glucose, fasting insulin, insulin resistance, postprandial insulin, body weight, BMI, waist circumference, and blood pressure | 12 weeks | 7.2g | Sequence generation: Low  Allocation concealment: Unclear  Blinding of participants: Unclear  Blinding of outcome: Unclear  Incomplete data: Unclear  Selective reporting: Low  Other: Low |
| Peterson 1987 **UK ^40^** | Randomised Crossover Trial with two relevant intervention arms | 16(6F) with T2. 60y. Duration 9y. BMI 27.3. HbA1c 11.3%. Diet and oral | 5g guar gum at breakfast and dinner or bread with 7.6g guar gum | Given white or wholemeal bread | HbA1c, fasting glucose, fasting insulin, body weight, total cholesterol, LDL, HDL, and triglycerides | 6 weeks | 6.1g or 6.4g by subgroup | Sequence generation: Unclear  Allocation concealment: Unclear  Blinding of participants: Unclear  Blinding of outcome: Unclear  Incomplete data: Unclear  Selective reporting: Low  Other: Low |
| Pick 1996 **Canada ^41^** | Randomised Crossover Trial | 8 with T2. 46y. BMI 27.6. Duration 2.1y. HbA1c 7%. Diet and oral | Given 8+ serves of bread with oat bran concentrate bread | Given 8+ serves white bread | Total cholesterol and triglycerides | 12 weeks | 14.8g | Sequence generation: Unclear  Allocation concealment: Unclear  Blinding of participants: Unclear  Blinding of outcome: Unclear  Incomplete data: Low  Selective reporting: Low  Other: Low |
| Roshnaravan 2017 **Iran ^42^** | Randomised Parallel Trial | 30 with T2 (17F).  Control: 52y. BMI 30.9.  Intervention: 51y. BMI 30.4. | 10g inulin powder and six capsules of starch powder | 10g starch powder and 6 capsules of starch powder | HbA1c, fasting glucose, fasting insulin, HOMA IR, body weight, BMI, waist circumference, total cholesterol, LDL, HDL, triglycerides, SBP, and DBP | 45 days | 10g | Sequence generation: Low  Allocation concealment: Unclear  Blinding of participants: Low  Blinding of outcome: Low  Incomplete data: Low  Selective reporting: Low  Other: Low |
| Sartore 2009 **Italy ^43^** | Non-randomised Parallel Trial | 40(13F) with T2. 20(7F) in the control: 60y. BMI 30.5. HbA1c 7.0%. 20(6F) in the intervention: 61y. BMI 30.1. HbA1c 6.8%.  Diet and oral | 3.5g psyllium at each main meal | Diet advice | HbA1c, fasting glucose, BMI, waist circumference, blood pressure, total cholesterol, LDL, HDL, and triglycerides | 8 weeks | 5.3g | Sequence generation: High  Allocation concealment: Unclear  Blinding of participants: Unclear  Blinding of outcome: Unclear  Incomplete data: Unclear  Selective reporting: Low  Other: Low |
| Stahl 1990 **Germany ^44^**  Not available in English | Randomised Crossover Trial | 10(3F) with T2. 64y. BMI 28.5. Diet and oral | 5g guar gum at each main meal | Placebo at each main meals | HbA1c, body weight, total cholesterol, LDL, HDL, and triglycerides | 3 months | 12g | Sequence generation: Unclear  Allocation concealment: High  Blinding of participants: Unclear  Blinding of outcome: Unclear  Incomplete data: Low  Selective reporting: Low  Other: Low |
| Uusitupa 1989 **Finland ^45^** | Randomised Parallel Trial | 39(26F) with T2. 19(14F) in the control: 58.7y. Duration 8.7y. 76.4kg. 20(12F) in the intervention: 60.1y. Duration 9.7y. 74.3kg. Diet and oral | 5g guar gum at each main meal | 5g placebo at each main meal | HbA1c, fasting glucose, body weight, blood pressure, total cholesterol, HDL, and triglycerides | 3 months | 12g | Sequence generation: Unclear  Allocation concealment: Unclear  Blinding of participants: Unclear  Blinding of outcome: Unclear  Incomplete data: Unclear  Selective reporting: Low  Other: Low |

* We identified a later publication (2018) from this author which we believe presents the same data. We believe this publication to be the earliest use of that data.

** We identified multiple publications from this research group with the same data. We believe this publication to be the earliest use of that data.

3. Giacco R, Parillo M, Rivellese AA, et al. Long-term dietary treatment with increased amounts of fiber-rich low-glycemic index natural foods improves blood glucose control and reduces the number of hypoglycemic events in type 1 diabetic patients. Diabetes care 2000; 23(10): 1461-6.

4. Venhaus A, Chantelau E. Self-selected unrefined and refined carbohydrate diets do not affect metabolic control in pump-treated diabetic patients. Diabetologia 1988; 31(3): 153-7.

5. Canfora EE, van der Beek CM, Hermes GD, et al. Supplementation of diet with galacto-oligosaccharides increases bifidobacteria, but not insulin sensitivity, in obese prediabetic individuals. Gastroenterology 2017; 153(1): 87-97. e3.

6. Daimon M, Oizumi T, Kato T, et al. Effect of beta-glucan enriched barley flour on impaired glucose tolerance and safety evaluation of the product. Japanese pharmacology and therapeutics 2011; 39(1): 101-8. Not available in English

7. Errazuriz I, Dube S, Slama M, et al. Randomized controlled trial of a MUFA or fiber-rich diet on hepatic fat in prediabetes. The Journal of Clinical Endocrinology & Metabolism 2017; 102(5): 1765-74.

8. Garcia A, Otto B, Reich S, et al. Arabinoxylan consumption decreases postprandial serum glucose, serum insulin and plasma total ghrelin response in subjects with impaired glucose tolerance. European journal of clinical nutrition 2007; 61(3): 334.

9. Kobayakawa A, Suzuki T, Ikami T, Saito M, Yabe D, Seino Y. Improvement of fasting plasma glucose level after ingesting moderate amount of dietary fiber in Japanese men with mild hyperglycemia and visceral fat obesity. Journal of dietary supplements 2013; 10(2): 129-41.

10. Lankinen M, Schwab U, Kolehmainen M, et al. Whole grain products, fish and bilberries alter glucose and lipid metabolism in a randomized, controlled trial: the Sysdimet study. PloS one 2011; 6(8): e22646.

11. Peterson CM, Beyl RA, Marlatt KL, Martin CK, Aryana KJ, Marco ML, et al. Effect of 12 wk of resistant starch supplementation on cardiometabolic risk factors in adults with prediabetes: a randomized controlled trial. The American journal of clinical nutrition. 2018;108(3):492-501.

12. Kang R, Kim M, Chae JS, Lee S-H, Lee JH. Consumption of whole grains and legumes modulates the genetic effect of the APOA5-1131C variant on changes in triglyceride and apolipoprotein AV concentrations in patients with impaired fasting glucose or newly diagnosed type 2 diabetes. Trials 2014; 15(1): 100.

13. Kim M, Jeung SR, Jeong T-S, Lee S-H, Lee JH. Replacing with whole grains and legumes reduces Lp-PLA2 activities in plasma and PBMCs in patients with prediabetes or T2D. Journal of lipid research 2014; 55(8): 1762-71.

14. Schwab U, Louheranta A, Törrönen A, Uusitupa M. Impact of sugar beet pectin and polydextrose on fasting and postprandial glycemia and fasting concentrations of serum total and lipoprotein lipids in middle-aged subjects with abnormal glucose metabolism. European journal of clinical nutrition 2006; 60(9): 1073.

15. Abutair AS, Naser IA, Hamed AT. Soluble fibers from psyllium improve glycemic response and body weight among diabetes type 2 patients (randomized control trial). Nutrition journal 2016; 15(1): 86.

16. Aliasgharzadeh A, Dehghan P, Gargari BP, Asghari-Jafarabadi M. Resistant dextrin, as a prebiotic, improves insulin resistance and inflammation in women with type 2 diabetes: a randomised controlled clinical trial. British Journal of Nutrition 2015; 113(2): 321-30.

17. Aro A, Uusitupa M, Voutilainen E, Hersio K, Korhonen T, Siitonen O. Improved diabetic control and hypocholesterolaemic effect induced by long-term dietary supplementation with guar gum in type 2 (insulin-independent) diabetes. Diabetologia 1981; 21(1): 29-33.

18. Babiker R, Elmusharaf K, Keogh MB, Saeed AM. Effect of Gum Arabic (Acacia Senegal) supplementation on visceral adiposity index (VAI) and blood pressure in patients with type 2 diabetes mellitus as indicators of cardiovascular disease (CVD): a randomized and placebo-controlled clinical trial. Lipids in health and disease 2018; 17(1): 56.

19. Babiker R, Elmusharaf K, Keogh MB, Banaga AS, Saeed AM. Metabolic effect of gum Arabic (Acacia Senegal) in patients with type 2 diabetes mellitus (T2DM): randomized, placebo controlled double blind trial. Functional Foods in Health and Disease 2017; 7(3): 222-34.

20. Baker P. Placebo‐controlled trial of guar in poorly controlled Type II diabetes. Practical Diabetes International 1988; 5(1): 36-8.

21. Calvo-Rubio MB, Montero FP, Campos LS, Barco CE, Ruiz JA, Tapia GB. Use of guar gum as a supplement to the usual diet in type 2 diabetes. A long-term study. Atencion primaria 1989; 6: 20-1, 4-5, 8-30.

22. Chuang L-M, Jou T, Yang W, et al. Therapeutic effect of guar gum in patients with non-insulin-dependent diabetes mellitus. Journal of the Formosan Medical Association 1992; 91(1): 15-9.

23. Cugnet-Anceau C, Nazare J-A, Biorklund M, et al. A controlled study of consumption of β-glucan-enriched soups for 2 months by type 2 diabetic free-living subjects. British journal of nutrition 2010; 103(3): 422-8.

24. Dall'Alba V, Silva FM, Antonio JP, et al. Improvement of the metabolic syndrome profile by soluble fibre–guar gum–in patients with type 2 diabetes: a randomised clinical trial. British Journal of Nutrition 2013; 110(9): 1601-10.

25. Dehghan P, Pourghassem Gargari B, Faghfoori Z, Salekzamani S, Jafarabadi M. Comparative effect of inulin and oligofructose-enriched inulin on glycemic indices and blood pressure in women with type 2 diabetes: a randomized clinical trial. ZUMS Journal 2014; 22(91): 25-38. Not available in English

26. Diaz J, Durruty P, Tapia J, et al. The effects of a dietary fiber (white lupine bran) in the treatment of non-insulin-dependent diabetes. Revista medica de Chile 1990; 118(1): 24-32. Not available in English

27. Feinglos MN, Gibb RD, Ramsey DL, Surwit RS, McRorie JW. Psyllium improves glycemic control in patients with type-2 diabetes mellitus. Bioactive Carbohydrates and Dietary Fibre 2013; 1(2): 156-61.

28. Feldheim W, Wisker E. Dietary fibre and type 2 diabetes mellitus. Forum of Nutrition; 56: 174-6.

29. Gargari BP, Namazi N, Khalili M, Sarmadi B, Jafarabadi MA, Dehghan P. Is there any place for resistant starch, as alimentary prebiotic, for patients with type 2 diabetes? Complementary therapies in medicine 2015; 23(6): 810-5.

30. Grunberger G, Jen KLC, Artiss JD. The benefits of early intervention in obese diabetic patients with FBCx™—a new dietary fibre. Diabetes/metabolism research and reviews 2007; 23(1): 56-62.

31. Hagander B, Asp N-G, Efendić S, Nilsson-Ehle P, Scherstén B. Dietary fiber decreases fasting blood glucose levels and plasma LDL concentration in noninsulin-dependent diabetes mellitus patients. The American journal of clinical nutrition 1988; 47(5): 852-8.

32. Hagander B, Asp N, Ekman R, Nilsson-Ehle P, Scherstén B. Dietary fibre enrichment, blood pressure, lipoprotein profile and gut hormones in NIDDM patients. European journal of clinical nutrition 1989; 43(1): 35-44.

33. Jenkins DJ, Kendall CW, Augustin LS, et al. Effect of wheat bran on glycemic control and risk factors for cardiovascular disease in type 2 diabetes. Diabetes care 2002; 25(9): 1522-8.

34. Karlander S, Armyr I, Efendic S. Metabolic effects and clinical value of beet fiber treatment in NIDDM patients. Diabetes research and clinical practice 1991; 11(2): 65-71.

35. Kondo K, Morino K, Nishio Y, et al. Fiber-rich diet with brown rice improves endothelial function in type 2 diabetes mellitus: A randomized controlled trial. PloS one 2017; 12(6): e0179869.

36. Lalor B, Bhatnagar D, Winocour P, et al. Placebo‐controlled trial of the effects of guar gum and metformin on fasting blood glucose and serum lipids in obese, type 2 diabetic patients. Diabetic medicine 1990; 7(3): 242-5.

37. Li X, Cai X, Ma X, et al. Short-and long-term effects of wholegrain oat intake on weight management and glucolipid metabolism in overweight type-2 diabetics: a randomized control trial. Nutrients 2016; 8(9): 549.

38. Moustafa TA, Kamel HS, El Malt MA. High dietary fibre intake (Talbina) as adjunct in the management of diabetic macular edema. Journal of Medical Sciences 2007; 7(1): 81-7.

39. Pedersen C, Gallagher E, Horton F, et al. Host-microbiome interactions in human type 2 diabetes following prebiotic fibre (galacto-oligosaccharide) intake. British Journal of Nutrition 2016; 116(11): 1869-77.

40. Peterson DB, Ellis PR, Baylis JM. Low dose guar in a novel food product: Improved metabolic control in non-insulin-dependent diabetes. Diabetic Medicine 1987; 4(2): 111-5.

41. Pick ME, Hawrysh ZJ, Gee MI, Toth E, Garg ML, Hardin RT. Oat bran concentrate bread products improve long-term control of diabetes: A pilot study. Journal of the American Dietetic Association 1996; 96(12): 1254-61.

42. Roshanravan N, Mahdavi R, Alizadeh E, Jafarabadi MA, Hedayati M, Ghavami A, et al. Effect of butyrate and inulin supplementation on glycemic status, lipid profile and glucagon-like peptide 1 level in patients with type 2 diabetes: A randomized double-blind, placebo- controlled trial. Hormone and Metabolic Research. 2017;49(11):886-91.

43. Sartore G, Reitano R, Barison A, et al. The effects of psyllium on lipoproteins in type II diabetic patients. European Journal of Clinical Nutrition 2009; 63(10): 1269-71.

44. Stahl M, Berger W. [Comparison of guar gum, wheat bran and placebo on carbohydrate and lipid metabolism in type II diabetics]. Schweizerische medizinische Wochenschrift 1990; 120(12): 402-8. Not available in English

45. Uusitupa M, Siitonen O, Savolainen K, Silvasti M, Penttila I, Parviainen M. Metabolic and nutritional effects of long-term use of guar gum in the treatment of noninsulin-dependent diabetes of poor metabolic control. American Journal of Clinical Nutrition 1989; 49(2): 345-51.

**
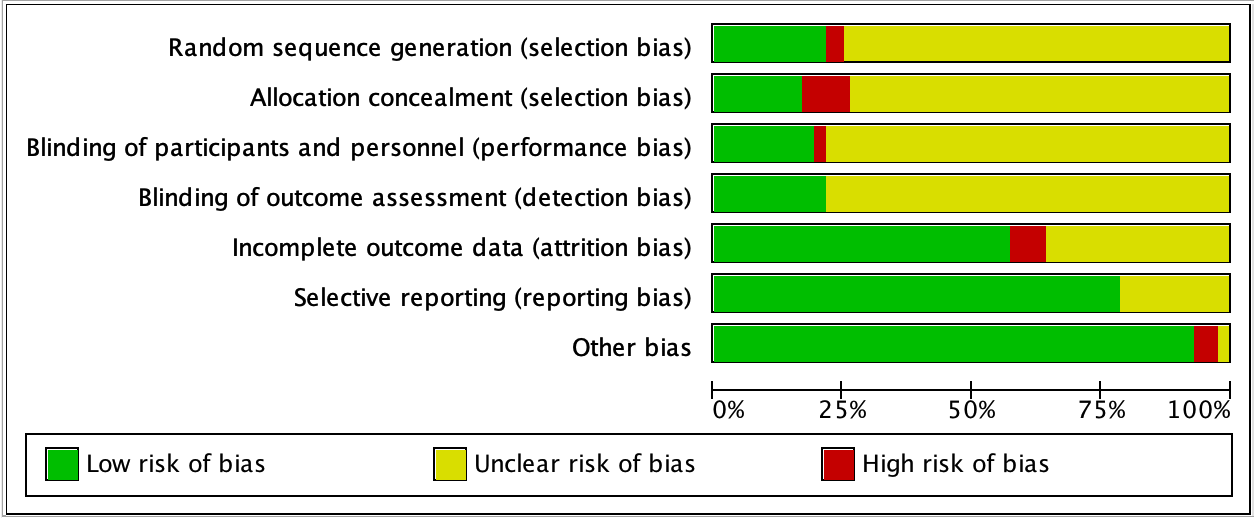
**

**S2 Fig A: Cochrane risk of bias tool summary for the trials identified as eligible for this review**
