# Supplementary material for: Dietary fibre and whole grains in diabetes management: Systematic review and meta-analyses
Source: PLoS Med. 2020 Mar 6;17(3):e1003053. doi: 10.1371/journal.pmed.1003053 (PMC7059907; doi:10.1371/journal.pmed.1003053)
Supplement: S3 Appendix — Fig A: Higher versus lower analysis for all-cause mortality. Data from 6 European countries in EPIC and EURODIAB cohort with random effects model. Fig B: Higher versus lower analysis for cardiovascular mortality. Data from 6 European countries in EPIC and EURODIAB cohort with random effects model. (DOCX) [file pmed.1003053.s003.docx]

**S3 Appendix.** Analyses for Fibre and Mortality

**Higher versus lower comparisons**

**S3 Fig A:** Higher versus lower analysis for all-cause mortality (data from six European countries in EPIC and EURODIAB cohort with random effects model. The higher quantile of fibre consumers is compared with the lowest quantile of fibre consumers, with a RR and 95%CI below 1.0 indicating an improvement in outcomes due to higher intakes. The per-country data from the EPIC cohort was obtained from the authors for this analysis.

Eggers test for publication bias p 0.877

Results of influence analyses: no one study influenced the pooled result

**S3 Fig B:** Higher versus lower analysis for cardiovascular mortality (data from six European countries in EPIC and EURODIAB cohort with random effects model. The higher quantile of fibre consumers is compared with the lowest quantile of fibre consumers, with a RR and 95%CI below 1.0 indicating an improvement in outcomes due to higher intakes. The per-country data from the EPIC cohort was obtained from the authors for this analysis.

Eggers test for publication bias p 0.481

Results of influence analyses: no one study influenced the pooled result

P value of meta regression of data for both outcomes to consider if the outcomes were different p 0.811.

Testing the linearity within the two stage random effects model

P value of Wald test for linearity of association between fibre intake and all-cause mortality p 0.0046

P value of Wald test for linearity of association between fibre intake and cardiovascular mortality p 0.1700
