# Supplementary material for: Dietary fibre and whole grains in diabetes management: Systematic review and meta-analyses
Source: PLoS Med. 2020 Mar 6;17(3):e1003053. doi: 10.1371/journal.pmed.1003053 (PMC7059907; doi:10.1371/journal.pmed.1003053)
Supplement: S4 Appendix — Fig A: Mean difference in HbA1c (mmol/mol) between intervention and control groups from trials of increasing fibre intakes. Table A: Univariate meta regression analyses to test for interaction. Fig B: Dose response curve for HbA1c (mmol/mol) when increasing fibre intakes accounting for baseline value when the data were available. (DOCX) [file pmed.1003053.s004.docx]

**S4 Appendix.** Analyses for fibre and HbA1c (mmol/mol)

**S4 Fig A:** Mean difference in HbA1c (mmol/mol) between intervention and control groups from trials of increasing fibre intakes.

Pooled mean difference was -2.00 mmol/mol (95%CI -3.30 to -0.71)

Egger’s test for publication bias p 0.252

Results of influence analyses: no one study influenced the pooled result

**S4 Table A:** Univariate meta regression analyses to test for interaction:

| **Continuous variables** | **P value** | **Global region** | **0.034** | Cochrane tool high bias | 0.191 |
| --- | --- | --- | --- | --- | --- |
| Trial size | 0.282 | Exclude by BMI | 0.355 | Wholegrain trial | 0.168 |
| Trial duration | 0.827 | **Dichotomous variables** | **P value** | Fibre incorporated into food | 0.053 |
| Baseline fibre intake when measured | **0.017** | **Weight controlled study** | **0.012** | Singular fibre type given | 0.128 |
| Fibre increase in intervention when measured | **0.013** | Exclude based on HbA1c | 0.349 | Imputed correlation coefficient | 0.328 |
| **Categorical variables** | **P value** | Exclude those aged over 65 | 0.189 | Viscosity | 0.785 |
| Type of diabetes | 0.792 | Exclude CVD/Renal participants | 0.801 | Solubility | 0.134 |
| Diabetes treatment | 0.689 | Parallel or crossover design | 0.126 |  |  |

Categorical variable ‘exclude by BMI’ tested for a difference in results between trials that excluded participants with a BMI <25, >35, or did not exclude based on BMI. Dichotomous variable ‘exclude based on HbA1c’ tested for a difference in results between trials that excluded participants based on their baseline HbA1c value.

These tests were undertaken to consider the robustness of the findings for HbA1c. These analyses indicated that beyond receiving the fibre intervention, other influences of the pooled result were: the baseline fibre intake when measured, the fibre increase in the intervention, the global region the study was conducted in, whether the trial were weight controlled. Results from subgroups for the categorical and dichotomous variables are shown in the HbA1c GRADE table below.

Given the importance of the amount of fibre and the baseline fibre intake in the overall relationship assessed, we have run dose response testing on the amount of fibre relative to baseline value.

**S4 Fig B:** Dose response curve for HbA1c (mmol/mol) when increasing fibre intakes accounting for baseline value when the data were available. The 95% confidence intervals are shown as dotted lines.

This curve was generated with data from 16 trials of 758 participants.
