# Supplementary material for: Dietary fibre and whole grains in diabetes management: Systematic review and meta-analyses
Source: PLoS Med. 2020 Mar 6;17(3):e1003053. doi: 10.1371/journal.pmed.1003053 (PMC7059907; doi:10.1371/journal.pmed.1003053)
Supplement: S5 Appendix — Fig A: Mean difference in fasting glucose (mmol/L) between intervention and control groups from trials of increasing fibre intakes. Table A: Univariate meta regression analyses as tests for interaction. Fig B: Dose response curve for fasting plasma glucose (mmol/mol) when increasing fibre intakes accounting for baseline value when known. (DOCX) [file pmed.1003053.s005.docx]

**S5 Appendix.** Analyses for fibre and fasting plasma glucose (mmol/L)

**S5 Fig A:** Mean difference in fasting glucose (mmol/L) between intervention and control groups from trials of increasing fibre intakes.

Pooled mean difference was -0.56 mmol/L (95%CI -0.73 to -0.38)

Egger’s test for publication bias p 0.207

Results of influence analyses: no one study influenced the pooled result

**S5 Table A:** Univariate meta regression analyses as tests for interaction:

| **Continuous variables** | **P value** | Global region | **<0.001** | Cochrane tool high bias | 0.213 |
| --- | --- | --- | --- | --- | --- |
| Trial size | 0.712 | Exclude by BMI | 0.796 | Wholegrain trial | 0.813 |
| Trial duration | 0.999 | **Dichotomous variables** | **P value** | Fibre incorporated into food | 0.687 |
| Baseline fibre intake when measured | **0.033** | Weight controlled study | 0.089 | Singular fibre type given | 0.975 |
| Fibre increase in intervention when measured | 0.393 | Exclude based on HbA1c | 0.637 | Imputed correlation coefficient | 0.090 |
| **Categorical variables** | **P value** | Exclude those aged over 65 | 0.092 | Viscosity | 0.980 |
| Type of diabetes | 0.498 | Exclude CVD/Renal participants | **0.009** | Solubility | 0.980 |
| Diabetes treatment | 0.182 | Parallel or crossover design | 0.796 |  |  |

These tests were undertaken to consider the robustness of the findings for fasting plasma glucose. These analyses indicated that beyond receiving the fibre intervention, other influences of the pooled result were: the baseline fibre intake when measured, and when trials excluded those with CVD or renal comorbidities. Results from subgroups for the categorical and dichotomous variables are shown in the fasting plasma glucose GRADE table below.

Given the importance of the amount of fibre and the baseline fibre intake in the overall relationship assessed, we have run dose response testing on the amount of fibre relative to baseline value.

**S5 Fig B:** Dose response curve for fasting plasma glucose (mmol/mol) when increasing fibre intakes accounting for baseline value when known. The 95% confidence intervals are shown as dotted lines.

This curve was generated with data from 18 trials of 979 participants.
