# Supplementary material for: Dietary fibre and whole grains in diabetes management: Systematic review and meta-analyses
Source: PLoS Med. 2020 Mar 6;17(3):e1003053. doi: 10.1371/journal.pmed.1003053 (PMC7059907; doi:10.1371/journal.pmed.1003053)
Supplement: S8 Appendix — Fig A: Mean difference in HDL cholesterol (mmol/L) between intervention and control groups from trials of increasing fibre intakes. Table A: Univariate meta regression analyses as tests for interaction. Fig B: Dose response curve for HDL cholesterol (mmol/L) when increasing fibre intakes. HDL, high-density lipoprotein. (DOCX) [file pmed.1003053.s008.docx]

**S8 Appendix.** Analyses for fibre and HDL cholesterol (mmol/L)

**S8 Fig A:** Mean difference in HDL cholesterol (mmol/L) between intervention and control groups from trials of increasing fibre intakes.

Pooled mean difference was 0.04 mmol/L (95%CI 0.01 to 0.07)

Egger’s test for publication bias p 0.978

Results of influence analyses: no one study influenced the pooled result

**S8 Table A:** Univariate meta regression analyses as tests for interaction:

| **Continuous variables** | **P value** | Global region | **<0.001** | Cochrane tool high bias | 0.051 |
| --- | --- | --- | --- | --- | --- |
| Trial size | 0.802 | Exclude by BMI | 0.338 | Wholegrain trial | 0.079 |
| Trial duration | 0.554 | **Dichotomous variables** | **P value** | Fibre incorporated into food | **0.018** |
| Baseline fibre intake when measured | 0.147 | Weight controlled study | 0.784 | Singular fibre type given | 0.520 |
| Fibre increase in intervention when measured | 0.964 | Exclude based on HbA1c | 0.265 | Imputed correlation coefficient | 0.163 |
| **Categorical variables** | **P value** | Exclude those aged over 65 | 0.192 | Viscosity | **0.043** |
| Type of diabetes | 0.428 | Exclude CVD/Renal participants | 0.651 | Solubility | 0.483 |
| Diabetes treatment | 0.790 | Parallel or crossover design | 0.346 |  |  |

These tests were undertaken to consider the robustness of the findings for HDL cholesterol. These analyses indicated that beyond receiving the fibre intervention, other influences of the pooled result were: the global region the trial was conducted in, whether the fibre was incorporated into food, and whether the fibre was viscous or not. Results from subgroups for the categorical and dichotomous variables are shown in the fasting plasma glucose GRADE table below.

**S8 Fig B:** Dose response curve for HDL cholesterol (mmol/L) when increasing fibre intakes. The 95% confidence intervals are shown as dotted lines.

This curve was generated with data from 24 trials of 1,329 participants.
