# Supplementary material for: Dietary fibre and whole grains in diabetes management: Systematic review and meta-analyses
Source: PLoS Med. 2020 Mar 6;17(3):e1003053. doi: 10.1371/journal.pmed.1003053 (PMC7059907; doi:10.1371/journal.pmed.1003053)
Supplement: S9 Appendix — Fig A: Mean difference in triglycerides (mmol/L) between intervention and control groups from trials of increasing fibre intakes. Table A: Univariate meta regression analyses as tests for interaction. Fig B: Dose response curve for triglycerides (mmol/L) when increasing fibre intakes. (DOCX) [file pmed.1003053.s009.docx]

**S9 Appendix.** Analyses for fibre and triglycerides (mmol/L)

**S9 Fig A:** Mean difference in triglycerides (mmol/L) between intervention and control groups from trials of increasing fibre intakes.

Pooled mean difference was -0.16 mmol/L (95%CI -0.23 to -0.09)

Egger’s test for publication bias p 0.958

Results of influence analyses: no one study influenced the pooled result

**S9 Table A:** Univariate meta regression analyses as tests for interaction:

| **Continuous variables** | **P value** | Global region | 0.495 | Cochrane tool high bias | 0.776 |
| --- | --- | --- | --- | --- | --- |
| Trial size | 0.524 | Exclude by BMI | 0.737 | Wholegrain trial | 0.111 |
| Trial duration | 0.983 | **Dichotomous variables** | **P value** | Fibre incorporated into food | 0.299 |
| Baseline fibre intake when measured | 0.334 | Weight controlled study | 0.155 | Singular fibre type given | 0.988 |
| Fibre increase in intervention when measured | 0.460 | Exclude based on HbA1c | 0.944 | Imputed correlation coefficient | 0.061 |
| **Categorical variables** | **P value** | Exclude those aged over 65 | 0.566 | Viscosity | 0.864 |
| Type of diabetes | 0.130 | Exclude CVD/Renal participants | 0.301 | Solubility | 0.255 |
| Diabetes treatment | 0.307 | Parallel or crossover design | 0.210 |  |  |

These tests were undertaken to consider the robustness of the findings for triglycerides. These analyses did not identify any factor beyond receiving the fibre intervention that might influence the pooled result.

**S9 Fig B:** Dose response curve for triglycerides (mmol/L) when increasing fibre intakes. The 95% confidence intervals are shown as dotted lines.

This curve was generated with data from 27 trials of 1,409 participants.
