# Supplementary material for: Dietary fibre and whole grains in diabetes management: Systematic review and meta-analyses
Source: PLoS Med. 2020 Mar 6;17(3):e1003053. doi: 10.1371/journal.pmed.1003053 (PMC7059907; doi:10.1371/journal.pmed.1003053)
Supplement: S11 Appendix — Fig A: Mean difference in BMI (kg/m2) between intervention and control groups from trials of increasing fibre intakes. Table A: Univariate meta regression analyses as tests for interaction. BMI, Body Mass Index. (DOCX) [file pmed.1003053.s011.docx]

**S11 Appendix.** Analyses for fibre and BMI (kg/m^2^)

**S11 Fig A:** Mean difference in BMI (kg/m^2^) between intervention and control groups from trials of increasing fibre intakes.

Pooled mean difference was -0.36 kg/m^2^ (95%CI -0.55 to -0.16)

Egger’s test for publication bias p 0.909

Results of influence analyses: no one study influenced the pooled result

**S11 Table A:** Univariate meta regression analyses as tests for interaction:

| **Continuous variables** | **P value** | Global region | 0.154 | Cochrane tool high bias | NA |
| --- | --- | --- | --- | --- | --- |
| Trial size | 0.782 | Exclude by BMI | 0.324 | Wholegrain trial | 0.459 |
| Trial duration | 0.936 | **Dichotomous variables** | **P value** | Fibre incorporated into food | 0.289 |
| Baseline fibre intake when measured | **0.043** | Weight controlled study | NA | Singular fibre type given | 0.598 |
| Fibre increase in intervention when measured | 0.850 | Exclude based on HbA1c | 0.585 | Imputed correlation coefficient | 0.944 |
| **Categorical variables** | **P value** | Exclude those aged over 65 | 0.377 | Viscosity | 0.915 |
| Type of diabetes | 0.503 | Exclude CVD/Renal participants | 0.627 | Solubility | 0.572 |
| Diabetes treatment | 0.284 | Parallel or crossover design | 0.454 |  |  |

These tests were undertaken to consider the robustness of the findings for BMI. These analyses indicated that beyond receiving the fibre intervention, other influences of the pooled result were: the baseline fibre intake when measured.

There were insufficient data to run dose response testing accounting for the baseline fibre intake values available.
