# Supplementary material for: Dietary fibre and whole grains in diabetes management: Systematic review and meta-analyses
Source: PLoS Med. 2020 Mar 6;17(3):e1003053. doi: 10.1371/journal.pmed.1003053 (PMC7059907; doi:10.1371/journal.pmed.1003053)
Supplement: S12 Appendix — Fig A: Mean difference in waist circumference (cm) between intervention and control groups from trials of increasing fibre intakes. Table A: Univariate meta regression analyses as tests for interaction. (DOCX) [file pmed.1003053.s012.docx]

**S12 Appendix.** Analyses for fibre and waist circumference (cm)

**S12 Fig A:** Mean difference in waist circumference (cm) between intervention and control groups from trials of increasing fibre intakes

Pooled mean difference was -1.42 cm (95%CI -2.63 to -0.21)

Egger’s test for publication bias p 0.498

Results of influence analyses: two studies (Sartore 2009 and Babiker 2018) were identified an influencing the pooled result. Without these two studies the pooled result was MD -1.59 cm (95%CI -3.07 to -0.10)

**S12 Table A:** Univariate meta regression analyses as tests for interaction:

| **Continuous variables** | **P value** | Global region | 0.072 | Cochrane tool high bias | NA |
| --- | --- | --- | --- | --- | --- |
| Trial size | 0.357 | Exclude by BMI | 0.426 | Wholegrain trial | NA |
| Trial duration | 0.986 | **Dichotomous variables** | **P value** | Fibre incorporated into food | NA |
| Baseline fibre intake when measured | 0.122 | Weight controlled study | NA | Singular fibre type given | 0.565 |
| Fibre increase in intervention when measured | NA | Exclude based on HbA1c | 0.459 | Imputed correlation coefficient | NA |
| **Categorical variables** | **P value** | Exclude those aged over 65 | 0.499 | Viscosity | 0.493 |
| Type of diabetes | 0.964 | Exclude CVD/Renal participants | 0.700 | Solubility | 0.098 |
| Diabetes treatment | 0.497 | Parallel or crossover design | NA |  |  |

These tests were undertaken to consider the robustness of the findings for waist circumference. These analyses did not identify any factor beyond receiving the fibre intervention that might influence the pooled result.

Insufficient data available to run any dose response testing.
