# Supplementary material for: Dietary fibre and whole grains in diabetes management: Systematic review and meta-analyses
Source: PLoS Med. 2020 Mar 6;17(3):e1003053. doi: 10.1371/journal.pmed.1003053 (PMC7059907; doi:10.1371/journal.pmed.1003053)
Supplement: S13 Appendix — Fig A: Mean difference in systolic blood pressure (mmHg) between intervention and control groups from trials of increasing fibre intakes. Table A: Univariate meta regression analyses as tests for interaction. Fig B: Dose response curve for systolic blood pressure (mmHg) when increasing fibre intakes. (DOCX) [file pmed.1003053.s013.docx]

**S13 Appendix.** Analyses for fibre and systolic blood pressure (mmHg)

**S13 Fig A:** Mean difference in systolic blood pressure (mmHg) between intervention and control groups from trials of increasing fibre intakes

Pooled mean difference was -1.9 mmHg (95%CI -4.9 to 1.1)

Egger’s test for publication bias p 0.972

Results of influence analyses: one study (Babiker 2018) influenced the pooled result. Without Babiker 2018 the pooled estimate was MD -1.5 mmHg (95%CI -4.6 to 1.7)

**S13 Table A:** Univariate meta regression analyses as tests for interaction:

| **Continuous variables** | **P value** | Global region | 0.186 | Cochrane tool high bias | 0.267 |
| --- | --- | --- | --- | --- | --- |
| Trial size | 0.728 | Exclude by BMI | 0.867 | Wholegrain trial | 0.556 |
| Trial duration | 0.888 | **Dichotomous variables** | **P value** | Fibre incorporated into food | 0.534 |
| Baseline fibre intake when measured | 0.975 | Weight controlled study | 0.436 | Singular fibre type given | 0.300 |
| Fibre increase in intervention when measured | 0.264 | Exclude based on HbA1c | 0.088 | Imputed correlation coefficient | 0.267 |
| **Categorical variables** | **P value** | Exclude those aged over 65 | 0.526 | Viscosity | 0.425 |
| Type of diabetes | 0.793 | Exclude CVD/Renal participants | 0.531 | Solubility | 0.180 |
| Diabetes treatment | 0.803 | Parallel or crossover design | 0.267 |  |  |

These tests were undertaken to consider the robustness of the findings for systolic blood pressure. These analyses did not identify any factor beyond receiving the fibre intervention that might influence the pooled result.

**S13 Fig B:** Dose response curve for systolic blood pressure (mmHg) when increasing fibre intakes. The 95% confidence intervals are shown as dotted lines.

This curve was generated with data from 11 trials of 561 participants.
