# Supplementary material for: Dietary fibre and whole grains in diabetes management: Systematic review and meta-analyses
Source: PLoS Med. 2020 Mar 6;17(3):e1003053. doi: 10.1371/journal.pmed.1003053 (PMC7059907; doi:10.1371/journal.pmed.1003053)
Supplement: S15 Appendix — Fig A: Standardised mean difference in CRP between intervention and control groups from trials of increasing fibre intakes. Table A: Univariate meta regression analyses as tests for interaction. CRP, C-reactive protein; SMD, standardised mean difference. (DOCX) [file pmed.1003053.s015.docx]

**S15 Appendix.** Analyses for fibre and C-reactive protein (SMD)

**S15 Fig A:** Standardised mean difference in CRP between intervention and control groups from trials of increasing fibre intakes

Pooled mean difference was SMD -2.8 (95%CI -4.5 to -1.1)

Egger’s test for publication bias p 0.081

Results of influence analyses: one study (Grunberger 2007) influenced the pooled result. Without Grunberger 2007 the pooled estimate was SMD -3.6 (95%CI -4.6 to -2.7)

**S15 Table A:** Univariate meta regression analyses as tests for interaction:

| **Continuous variables** | **P value** | Global region | 0.700 | Cochrane tool high bias | 0.999 |
| --- | --- | --- | --- | --- | --- |
| Trial size | 0.195 | Exclude by BMI | 0.545 | Wholegrain trial | 0.293 |
| Trial duration | 0.268 | **Dichotomous variables** | **P value** | Fibre incorporated into food | 0.353 |
| Baseline fibre intake when measured | 0.558 | Weight controlled study | 0.999 | Singular fibre type given | 0.353 |
| Fibre increase in intervention when measured | NA | Exclude based on HbA1c | **0.009** | Imputed correlation coefficient | 0.999 |
| **Categorical variables** | **P value** | Exclude those aged over 65 | 0.094 | Viscosity | NA |
| Type of diabetes | 0.510 | Exclude CVD/Renal participants | 0.999 | Solubility | 0.195 |
| Diabetes treatment | 0.994 | Parallel or crossover design | 0.999 |  |  |

These tests were undertaken to consider the robustness of the findings for CRP. These analyses indicated that beyond receiving the fibre intervention, other influences of the pooled result were: the exclusion of participants based on HbA1c values. Results from subgroups for the categorical and dichotomous variables are shown in the fasting plasma glucose GRADE table below.

Insufficient data available to run any dose response testing.
