# Supplementary material for: Dietary fibre and whole grains in diabetes management: Systematic review and meta-analyses
Source: PLoS Med. 2020 Mar 6;17(3):e1003053. doi: 10.1371/journal.pmed.1003053 (PMC7059907; doi:10.1371/journal.pmed.1003053)
Supplement: S16 Appendix — Fig A: Standardised mean difference in fasting insulin between intervention and control groups from trials of increasing fibre intakes. Table A: Univariate meta regression analyses as tests for interaction. SMD, standardised mean difference. (DOCX) [file pmed.1003053.s016.docx]

**S16 Appendix.** Analyses for fibre and fasting plasma insulin (SMD)

**S16 Fig A:** Standardised mean difference in fasting insulin between intervention and control groups from trials of increasing fibre intakes

Pooled mean difference was SMD -2.03 (95%CI -2.92 to -1.13)

Egger’s test for publication bias p 0.011. Trim and fill analysis changed the observed SMD to -2.48 (95%CI -3.56 to -1.42).

Results of influence analyses: one study (Laniken 2011) as significantly influencing the pooled result. The standardised mean difference after removing Laniken 2011 was -2.19 (-3.07 to -1.32).

**S16 Table A:** Univariate meta regression analyses as tests for interaction:

| **Continuous variables** | **P value** | Global region | **0.011** | Cochrane tool high bias | 0.516 |
| --- | --- | --- | --- | --- | --- |
| Trial size | 0.783 | Exclude by BMI | 0.088 | Wholegrain trial | 0.196 |
| Trial duration | 0.350 | **Dichotomous variables** | **P value** | Fibre incorporated into food | 0.163 |
| Baseline fibre intake when measured | 0.187 | Weight controlled study | 0.476 | Singular fibre type given | 0.542 |
| Fibre increase in intervention when measured | 0.882 | Exclude based on HbA1c | 0.511 | Imputed correlation coefficient | 0.814 |
| **Categorical variables** | **P value** | Exclude those aged over 65 | 0.546 | Viscosity | 0.189 |
| Type of diabetes | 0.544 | Exclude CVD/Renal participants | 0.849 | Solubility | 0.447 |
| Diabetes treatment | 0.990 | Parallel or crossover design | 0.865 |  |  |

These tests were undertaken to consider the robustness of the findings for fasting plasma insulin. These analyses indicated that beyond receiving the fibre intervention, other influences of the pooled result were: the global region the trial was conducted in.

Results from subgroups for the categorical and dichotomous variables are shown in the fasting plasma glucose GRADE table below.
