# Supplementary material for: Dietary fibre and whole grains in diabetes management: Systematic review and meta-analyses
Source: PLoS Med. 2020 Mar 6;17(3):e1003053. doi: 10.1371/journal.pmed.1003053 (PMC7059907; doi:10.1371/journal.pmed.1003053)
Supplement: S17 Appendix — Fig A: Mean difference in HOMA IR (mg/dL) between intervention and control groups from trials of increasing fibre intakes. Table A: Univariate meta regression analyses as tests for interaction. Fig B: Dose response curve for HOMA IR (mg/dL) when increasing fibre intakes. HOMA IR, homeostatic model assessment of insulin resistance. (DOCX) [file pmed.1003053.s017.docx]

**S17 Appendix.** Analyses for fibre and HOMA IR (mg/dL)

**S17 Fig A:** Mean difference in HOMA IR (mg/dL) between intervention and control groups from trials of increasing fibre intakes

Pooled mean difference was -1.24 mg/dL (95%CI -1.72 to -0.76)

Egger’s test for publication bias p 0.219

Results of influence analyses: one study (Laniken 2011) as significantly influencing the pooled result. The mean difference after removing Laniken 2011 was -1.41 (-1.91 to -0.92).

**S17 Table A:** Univariate meta regression analyses as tests for interaction:

| **Continuous variables** | **P value** | Global region | 0.060 | Cochrane tool high bias | NA |
| --- | --- | --- | --- | --- | --- |
| Trial size | 0.463 | Exclude by BMI | 0.810 | Wholegrain trial | 0.133 |
| Trial duration | 0.274 | **Dichotomous variables** | **P value** | Fibre incorporated into food | 0.133 |
| Baseline fibre intake when measured | 0.878 | Weight controlled study | NA | Singular fibre type given | 0.066 |
| Fibre increase in intervention when measured | 0.772 | Exclude based on HbA1c | 0.579 | Imputed correlation coefficient | NA |
| **Categorical variables** | **P value** | Exclude those aged over 65 | 0.502 | Viscosity | 0.054 |
| Type of diabetes | 0.609 | Exclude CVD/Renal participants | 0.162 | Solubility | 0.228 |
| Diabetes treatment | 0.455 | Parallel or crossover design | NA |  |  |

These tests were undertaken to consider the robustness of the findings for HOMA IR. These analyses did not identify any factor beyond receiving the fibre intervention that might influence the pooled result.

**S17 Fig B:** Dose response curve for HOMA IR (mg/dL) when increasing fibre intakes. The 95% confidence intervals are shown as dotted lines.

This curve was generated with data from 9 trials of 581 participants.
