# Supplementary material for: Dietary fibre and whole grains in diabetes management: Systematic review and meta-analyses
Source: PLoS Med. 2020 Mar 6;17(3):e1003053. doi: 10.1371/journal.pmed.1003053 (PMC7059907; doi:10.1371/journal.pmed.1003053)
Supplement: S18 Appendix — Table A: Do greater intakes of total dietary fibre reduce the risk of all-cause and CVD mortality for adults with type1 or type 2 diabetes? Table B: What is the effect of increasing dietary fibre intakes on HbA1c (mmol/mol) in diabetes management? Table C: What is the effect of increasing dietary fibre intakes on fasting plasma glucose (mmol/L) in diabetes management? Table D: What is the effect of increasing dietary fibre intakes on total cholesterol (mmol/L) in diabetes management? Table E: What is the effect of increasing dietary fibre intakes on LDL cholesterol (mmol/L) in diabetes management? Table F: What is the effect of increasing dietary fibre intakes on HDL cholesterol (mmol/L) in diabetes management? Table G: What is the effect of increasing dietary fibre intakes on triglycerides (mmol/L) in diabetes management? Table H: What is the effect of increasing dietary fibre intakes on body weight (kg) in diabetes management? Table I: What is the effect of increasing dietary fibre intakes on BMI in diabetes management? Table J: What is the effect of increasing dietary fibre intakes on waist circumference (cm) in diabetes management? Table K: What is the effect of increasing dietary fibre intakes on fasting plasma insulin (standardised mean difference) in diabetes management? Table L: What is the effect of increasing dietary fibre intakes on HOMA IR (mg/dL) in diabetes management? Table M: What is the effect of increasing dietary fibre intakes on systolic blood pressure (mmHg) in diabetes management? Table N: What is the effect of increasing dietary fibre intakes on diastolic blood pressure (mmHg) in diabetes management? Table O: What is the effect of increasing dietary fibre intakes on C-reactive protein in diabetes management? BMI, Body Mass Index; CVD, Cardiovascular disease; GRADE, Grading of Recommendations Assessment, Development and Evaluation; HDL, high-denisty lipoprotein; HOMA IR, homeostatic model assessment of insulin resistance; LDL, low-density lipop [file pmed.1003053.s018.docx]

**S18 Appendix.** GRADE tables

Authors: Andrew N Reynolds, Ashley Akerman, Jim Mann

Date: 31 May 2019

Question: Do greater intakes of total dietary fibre reduce the risk of all-cause and CVD mortality for adults with type1 or type 2 diabetes?

Setting: Data from adults included in prospective observational studies, searches run January 2019.

S18 Table A

| **Certainty assessment** | | | | | | | **№ of patients** | | **Effect** | | **Certainty** |
| --- | --- | --- | --- | --- | --- | --- | --- | --- | --- | --- | --- |
| **№ of studies** | **Study design** | **Risk of bias** | **Inconsistency** | **Indirectness** | **Imprecision** | **Other considerations** | **Person years** | **Incidence/ number of people** | **Relative (95% CI)** | **Absolute (95% CI)** |  |
| What is the effect of consuming greater versus lower amounts of total fibre on all-cause mortality? (weighted mean follow up time 8.8 years) | | | | | | | | | | | |
| 2 | prospective studies | not serious ^a^ | not serious | not serious | not serious | dose response gradient | 72,355 | 837/8300 | **RR 0·55** (0·35 to 0·86) | **45 fewer per 1,000** (from 14 fewer to 66 fewer) | ⨁⨁⨁ MODERATE |
| What is the effect of consuming greater versus lower amounts of total fibre on CVD mortality? (weighted mean follow up time 8.8 years) | | | | | | | | | | | |
| 2 | prospective studies | not serious ^a^ | not serious | not serious | not serious | none | 72,355 | 321/8300 | **RR 0·61** (0·26 to 1·42) | **15 fewer per 1,000** (from 29 fewer to 16 more) | ⨁⨁ LOW |

**CI:** Confidence interval; **MD:** Mean difference.

#### Explanations

a. Although the data were obtained from only two publications, both publications were of multi-centre studies throughout Europe. As such, their results are perceived to be more reflective of a broader population than what would normally be obtained from two single-centre studies. Estimates use the per-country effect size estimates wherever possible.

**Authors**: Andrew N Reynolds, Ashley Akerman, Jim Mann

**Date**: 8 December 2019

**Question**: What is the effect of increasing dietary fibre intakes on HbA1c (mmol/mol) in diabetes management?

**Setting**: Adults with prediabetes, type 1 diabetes, or type 2 diabetes participating in intervention trials, searches run January 2019.

S18 Table B

| **Certainty assessment** | | | | | | | | **№ of patients** | | **Effect** | **Certainty** |
| --- | --- | --- | --- | --- | --- | --- | --- | --- | --- | --- | --- |
| **Subgroup** | **№ of trials** | **Study design** | **Risk of bias** | **Inconsistency** | **Indirectness** | **Imprecision** | **Other** | **Intervention** | **Control** | **Mean difference (95% CI)** |  |
| Overall | 33 | controlled trials | not serious ^a,b^ | serious ^c^ | not serious | not serious | none | 815 | 738 | 2.00 mmol/mol lower (3.30 lower to 0.71 lower) | ⨁⨁⨁ MODERATE |
| Mean difference in HbA1c where the weight of participants was controlled or not controlled over trial duration. | | | | | | | | | | | |
| Weight controlled | 5 | controlled trials | not serious | serious ^d^ | not serious | serious ^e^ | none | 122 | 101 | 1.26 mmol/mol higher (0.15 lower to 2.68 higher) | ⨁⨁ LOW |
| Not weight controlled | 28 | controlled trials | not serious | serious ^f^ | not serious | not serious | none | 693 | 637 | 2.67 mmol/mol lower (4.18 lower to 1.16 lower) | ⨁⨁⨁ MODERATE |
| Mean difference in HbA1c by global region | | | | | | | | | | | |
| Europe | 15 | controlled trials | not serious | serious ^g^ | not serious | not serious | none | 298 | 242 | 0.01 mmol/mol higher (1.30 lower to 1.32 higher) | ⨁⨁⨁ MODERATE |
| North America | 4 | controlled trials | not serious | serious ^h^ | not serious | serious ^e^ | none | 101 | 88 | 1.69 mmol/mol lower (4.03 lower to 0.65 higher) | ⨁⨁ LOW |
| Asia | 5 | controlled trials | not serious | serious ^i^ | not serious | not serious | none | 174 | 174 | 1.98 mmol/mol lower (4.51 lower to 0.44 higher) | ⨁⨁⨁ MODERATE |
| Middle East | 6 | controlled trials | not serious | serious ^i^ | not serious | not serious | none | 157 | 152 | 5.63 mmol/mol lower (8.63 lower to 2.63 lower) | ⨁⨁⨁ MODERATE |

**CI:** Confidence interval; **MD:** Mean difference. Subgroups with the data of two or less trials are not shown.

#### Explanations

a. Eggers p for possible publication bias 0.252 indicated low risk.

b. Influence analysis of the standardised mean results of these trials did not identify any one study that significantly influenced the pooled result.

c. Subgroups are shown in this GRADE table where a univariate meta regression analysis identified a difference in pooled results due to an identified difference between trials.

d. Initial heterogeneity as measured by I^2^ was 90.1%

e. The number of participants in this pooled estimate failed to meet the optimal information size necessary to avoid risk of imprecision.

f. Initial heterogeneity as measured by I^2^ was 98.8%.

g. Initial heterogeneity as measured by I^2^ was 88.6%.

h. Initial heterogeneity as measured by I^2^ was 97.9%.

i. Initial heterogeneity as measured by I^2^ was 98.5%.

**Authors**: Andrew N Reynolds, Ashley Akerman, Jim Mann

**Date**: 8 December 2019

**Question**: What is the effect of increasing dietary fibre intakes on fasting plasma glucose (mmol/L) in diabetes management?

**Setting**: Adults with prediabetes, type 1 diabetes, or type 2 diabetes participating in intervention trials, searches run January 2019.

S18 Table C

| **Certainty assessment** | | | | | | | | **№ of patients** | | **Effect** | **Certainty** |
| --- | --- | --- | --- | --- | --- | --- | --- | --- | --- | --- | --- |
| **Subgroup** | **№ of trials** | **Study design** | **Risk of bias** | **Inconsistency** | **Indirectness** | **Imprecision** | **Other** | **Intervention** | **Control** | **Mean difference (95% CI)** |  |
| Overall | 34 | controlled trials | not serious ^a,b^ | serious ^c^ | not serious | not serious | dose response gradient | 936 | 871 | 0.56 mmol/L lower (0.73 lower to 0.38 lower) | ⨁⨁⨁⨁ HIGH |
| Mean difference in FPG (mmol/L) by whether participants with CVD or renal issues were included or not | | | | | | | | | | | |
| Included participants with CVD/renal issues | 15 | controlled trials | not serious | serious ^d^ | not serious | not serious | none | 313 | 277 | 0.91 mmol/L lower (1.46 lower to 0.36 lower) | ⨁⨁⨁ MODERATE |
| Excluded Participants with CVD/renal issues | 19 | controlled trials | not serious | serious ^e^ | not serious | not serious | none | 623 | 594 | 0.26 lower (0.46 lower to 0.05 lower) | ⨁⨁⨁ MODERATE |
| Mean difference in FPG (mmol/L) by global region | | | | | | | | | | | |
| Europe | 21 | controlled trials | not serious | serious ^f^ | not serious | not serious | none | 332 | 293 | 0.26 lower  (0.45 lower to 0.07 lower) | ⨁⨁⨁ MODERATE |
| North America | 3 | controlled trials | not serious | serious ^g^ | not serious | serious ^h^ | none | 81 | 61 | 1.00 lower  (1.69 lower to 0.32 lower) | ⨁⨁ LOW |
| Asia | 8 | controlled trials | not serious | serious ^i^ | not serious | not serious | none | 296 | 298 | 0.54 lower  (0.83 lower to 0.24 lower) | ⨁⨁⨁ MODERATE |
| Middle East | 6 | controlled trials | not serious | serious ^j^ | not serious | not serious ^h^ | none | 142 | 137 | 1.11 lower  (1.58 lower to 0.64 lower) | LOW |

**CI:** Confidence interval; **MD:** Mean difference. Subgroups with the data of two or less trials are not shown.

#### Explanations

a. Eggers p for possible publication bias 0.207 indicated low risk.

b. Influence analysis of the standardised mean results of these trials did not identify any one study that significantly influenced the pooled result.

c. Subgroups are shown in this GRADE table where a univariate meta regression analysis identified a difference in pooled results due to an underlying difference between trials.

d. Initial heterogeneity as measured by I^2^ was 99.1%.

e. Initial heterogeneity as measured by I^2^ was 99.1%.

f. Initial heterogeneity as measured by I^2^ was 96.0%.

g. Initial heterogeneity as measured by I^2^ was 97.2%.

h. The number of participants in this pooled estimate failed to meet the optimal information size necessary to avoid risk of imprecision.

i. Initial heterogeneity as measured by I^2^ was 99.4%.

j. Initial heterogeneity as measured by I^2^ was 98.7%.

**Authors**: Andrew N Reynolds, Ashley Akerman, Jim Mann

**Date**: 8 December 2019

**Question**: What is the effect of increasing dietary fibre intakes on total cholesterol (mmol/L) in diabetes management?

**Setting**: Adults with prediabetes, type 1 diabetes, or type 2 diabetes participating in intervention trials, searches run January 2019.

S18 Table D

| **Certainty assessment** | | | | | | | | **№ of patients** | | **Effect** | **Certainty** |
| --- | --- | --- | --- | --- | --- | --- | --- | --- | --- | --- | --- |
| **Subgroup** | **№ of trials** | **Study design** | **Risk of bias** | **Inconsistency** | **Indirectness** | **Imprecision** | **Other** | **Intervention** | **Control** | **Mean difference (95% CI)** |  |
| Overall | 27 | controlled trials | not serious ^a,b^ | serious ^c^ | not serious | not serious | dose response gradient | 662 | 605 | 0.34 mmol/L lower (0.46 lower to 0.22 lower) | ⨁⨁⨁⨁ HIGH |

**CI:** Confidence interval; **MD:** Mean difference

#### Explanations

a. Eggers p for possible publication bias 0.329 indicated low risk.

b. Influence analysis of the standardised mean results of these trials did not identify any one study that significantly influenced the pooled result.

c. Univariate meta regression analysis did not identify a difference in pooled results due to an any underlying difference between trials beyond a change in fibre intake.

**Author**: Andrew N Reynolds, Ashley Akerman, Jim Mann

**Date**: 8 December 2019

**Question**: What is the effect of increasing dietary fibre intakes on LDL cholesterol (mmol/L) in diabetes management?

**Setting**: Adults with prediabetes, type 1 diabetes, or type 2 diabetes participating in intervention trials, searches run January 2019.

S18 Table E

| **Certainty assessment** | | | | | | | | **№ of patients** | | **Effect** | **Certainty** |
| --- | --- | --- | --- | --- | --- | --- | --- | --- | --- | --- | --- |
| **Subgroup** | **№ of trials** | **Study design** | **Risk of bias** | **Inconsistency** | **Indirectness** | **Imprecision** | **Other** | **Intervention** | **Control** | **Mean difference (95% CI)** |  |
| Overall | 21 | controlled trials | not serious ^a,b^ | serious ^c^ | not serious | not serious | dose response gradient | 559 | 512 | 0.17 mmol/L lower (0.27 lower to 0.08 lower) | ⨁⨁⨁⨁ HIGH |

**CI:** Confidence interval; **MD:** Mean difference

#### Explanations

#### a. Eggers p for possible publication bias 0.721 indicated low risk.

b. Influence analysis of the standardised mean results of these trials did not identify any one study that significantly influenced the pooled result.

c. Univariate meta regression analysis did not identify a difference in pooled results due to an any underlying difference between trials beyond a change in fibre intake.

**Authors**: Andrew N Reynolds, Ashley Akerman, Jim Mann

**Date**: 8 December 2019

**Question**: What is the effect of increasing dietary fibre intakes on HDL cholesterol (mmol/L) in diabetes management?

**Setting**: Adults with prediabetes, type 1 diabetes, or type 2 diabetes participating in intervention trials, searches run January 2019.

S18 Table F

| **Certainty assessment** | | | | | | | | **№ of patients** | | **Effect** | **Certainty** |
| --- | --- | --- | --- | --- | --- | --- | --- | --- | --- | --- | --- |
| **Subgroups** | **№ of trials** | **Study design** | **Risk of bias** | **Inconsistency** | **Indirectness** | **Imprecision** | **Other** | **Intervention** | **Control** | **Mean difference (95% CI)** |  |
| Overall | 25 | controlled trials | serious ^a^ | not serious ^b^ | not serious | not serious | none | 722 | 666 | 0.04 mmol/L higher (0.01 higher to 0.07 higher) | ⨁⨁⨁ MODERATE |
| Mean difference in HDL cholesterol (mmol/L) by fibre viscosity | | | | | | | | | | | |
| Viscous fibre | 11 | controlled trials | not serious | serious ^c^ | not serious | not serious | none | 285 | 250 | 0.02 mmol/L higher (0.01 lower to 0.04 higher) | ⨁⨁⨁ MODERATE |
| Non viscous fibre | 6 | controlled trials | not serious | serious ^d^ | not serious | serious ^e^ | none | 136 | 114 | 0.11 mmol/L higher (0.03 higher to 0.18 higher) | ⨁⨁ LOW |
| Mean differences in HDL cholesterol (mmol/L) by area of origin | | | | | | | | | | | |
| Europe | 12 | controlled trials | not serious | serious ^f^ | not serious | not serious | none | 264 | 210 | 0.01 mmol/L higher (0.01 lower to 0.04 higher) | ⨁⨁⨁ MODERATE |
| Asia | 5 | controlled trials | not serious | serious ^g^ | not serious | not serious | none | 251 | 252 | 0.01 mmol/L lower (0.04 lower to 0.02 higher) | ⨁⨁⨁ MODERATE |
| Middle east | 3 | controlled trials | not serious | serious ^h^ | not serious | serious ^e^ | none | 70 | 69 | 0.13 mmol/L higher  (0.01 higher to 0.25 higher) | ⨁⨁ LOW |
| Mean differences in HDL cholesterol (mmol/L) when fibre is provided in a food or provided alone as supplement | | | | | | | | | | | |
| Fibre in a food | 11 | controlled trials | not serious | serious ^i^ | not serious | not serious | none | 368 | 352 | 0.00 mmol/L  (0.03 lower to 0.02 higher) | ⨁⨁⨁ MODERATE |
| Fibre alone | 15 | controlled trials | not serious | serious ^j^ | not serious | not serious | none | 354 | 314 | 0.07 mmol/L higher  (0.03 higher to 0.11 higher) | ⨁⨁⨁ MODERATE |

**CI:** Confidence interval; **MD:** Mean difference

#### Explanations

a. Eggers p for possible publication bias was 0.978 suggesting low risk.

b. Subgroups are shown in this GRADE table where a univariate meta regression analysis identified a difference in pooled results due to an underlying difference between trials.

c. Initial heterogeneity as measured by I^2^ was 82.2%.

d. Initial heterogeneity as measured by I^2^ was 97.7%.

e. The number of participants in this pooled estimate failed to meet the optimal information size necessary to avoid risk of imprecision.

f. Initial heterogeneity as measured by I^2^ was 76.9%.

g. Initial heterogeneity as measured by I^2^ was 96.3%.

h. Initial heterogeneity as measured by I^2^ was 93.8%.

i. Initial heterogeneity as measured by I^2^ was 97.0%.

j. Initial heterogeneity as measured by I^2^ was 98.1%.

**Authors**: Andrew N Reynolds, Ashley Akerman, Jim Mann

**Date**: 8 December 2019

**Question**: What is the effect of increasing dietary fibre intakes on triglycerides (mmol/L) in diabetes management?

**Setting**: Adults with prediabetes, type 1 diabetes, or type 2 diabetes participating in intervention trials, searches run January 2019.

S18 Table G

| **Certainty assessment** | | | | | | | | **№ of patients** | |  | **Certainty** |
| --- | --- | --- | --- | --- | --- | --- | --- | --- | --- | --- | --- |
| **Subgroup** | **№ of trials** | **Study design** | **Risk of bias** | **Inconsistency** | **Indirectness** | **Imprecision** | **Other** | **Intervention** | **Control** | **Mean differences (95% CI)** |  |
| Overall | 28 | controlled trials ^a^ | not serious ^b,c^ | serious ^d,e^ | not serious | not serious | none | 760 | 708 | 0.16 mmol/L lower (0.23 lower to 0.09 lower) | ⨁⨁⨁ MODERATE |

**CI:** Confidence interval; **MD:** Mean difference

#### Explanations

a. Kondo 2017 was removed from this analysis as the reported values were outside what is biologically plausible.

b. Influence analysis of the standardised mean results of these trials did not identify any study that significantly influenced the pooled result.

c. Eggers p for possible publication bias was 0.958 suggesting low risk.

d. An initial univariate meta regression identified that our imputed correlation coefficients were too conservative, so we increased the imputed values by 10%.

e. Initial heterogeneity as measured by I^2^ was 97.7%.

**Authors**: Andrew N Reynolds, Ashley Akerman, Jim Mann

**Date**: 8 December 2019

**Question**: What is the effect of increasing dietary fibre intakes on body weight (kg) in diabetes management?

**Setting**: Adults with prediabetes, type 1 diabetes, or type 2 diabetes participating in intervention trials, searches run January 2019.

S18 Table H

| **Certainty assessment** | | | | | | | | **№ of patients** | | **Effect** | **Certainty** |
| --- | --- | --- | --- | --- | --- | --- | --- | --- | --- | --- | --- |
| **Subgroup** | **№ of trials** | **Study design** | **Risk of bias** | **Inconsistency** | **Indirectness** | **Imprecision** | **Other** | **Intervention** | **Control** | **Mean difference (95% CI)** |  |
| Overall | 18 | controlled trials ^a^ | not serious ^b,c^ | serious ^d,e^ | not serious | not serious | none | 455 | 422 | 0.56 kg lower 0.98 lower to 0.13 lower) | ⨁⨁⨁ MODERATE |

**CI:** Confidence interval; **MD:** Mean difference

#### Explanations

a. Trials that controlled the weight of the participants across the study duration were removed from these analyses.

b. Eggers p for possible publication bias 0.767 indicated low risk.

c. Influence analysis of the standardised mean results of these trials indicated that two studies (Li 2016 and Babiker 2017) significantly influenced the pooled result. Without these two studies the pooled mean difference is: -0.49 (-0.95 to -0.02).

d. Univariate meta regression analysis did not identify a difference in pooled results due to an any underlying difference between trials beyond a change in fibre intake.

e. Initial heterogeneity as measured by I^2^ was 98.2%.

**Authors**: Andrew N Reynolds, Ashley Akerman, Jim Mann

**Date**: 8 December 2019

**Question**: What is the effect of increasing dietary fibre intakes on BMI in diabetes management?

**Setting**: Adults with prediabetes, type 1 diabetes, or type 2 diabetes participating in intervention trials, searches run January 2019.

S18 Table I

| **Certainty assessment** | | | | | | | | **№ of patients** | | **Effect** | **Certainty** |
| --- | --- | --- | --- | --- | --- | --- | --- | --- | --- | --- | --- |
| **Subgroup** | **№ of trials** | **Study design** | **Risk of bias** | **Inconsistency** | **Indirectness** | **Imprecision** | **Other** | **Intervention** | **Control** | **Mean differences (95% CI)** |  |
| Overall | 14 | controlled trials ^a^ | not serious ^b,c^ | serious ^d^ | not serious | not serious | none ^e^ | 382 | 381 | 0.36 lower (0.55 lower to 0.16 lower) | ⨁⨁⨁ MODERATE |

**CI:** Confidence interval; **MD:** Mean difference

#### Explanations

a. Trials where the body weight of participants was controlled throughout the study were removed from these analyses.

b. Eggers p for possible publication bias 0.909 indicated low risk.

c. Influence analysis of the standardised mean results of these trials did not identify any study that significantly influenced the pooled result.

d. Initial heterogeneity as measured by I^2^ was 97.4%

e. Although a continuous variable meta regression identified that baseline fibre intake was an important determinant of overall response, there were insufficient data available to consider dose response testing with baseline fibre intakes.

**Authors**: Andrew N Reynolds, Ashley Akerman, Jim Mann

**Date**: 8 December 2019

**Question**: What is the effect of increasing dietary fibre intakes on waist circumference (cm) in diabetes management?

**Setting**: Adults with prediabetes, type 1 diabetes, or type 2 diabetes participating in intervention trials, searches run January 2019.

S18 Table J

| **Certainty assessment** | | | | | | | | **№ of patients** | | **Effect** | **Certainty** |
| --- | --- | --- | --- | --- | --- | --- | --- | --- | --- | --- | --- |
| **Subgroup** | **№ of trials** | **Study design** | **Risk of bias** | **Inconsistency** | **Indirectness** | **Imprecision** | **Other** | **Intervention** | **Control** | **Mean difference (95% CI)** |  |
| Overall | 8 | controlled trials ^a^ | not serious ^b,c^ | serious ^d^ | not serious | not serious | none | 178 | 171 | 1.42 cm lower (2.63 lower to 0.21 lower) | ⨁⨁⨁ MODERATE |

**CI:** Confidence interval; **MD:** Mean difference

#### Explanations

a. Trials that controlled the weight of the participants across the study duration were removed from these analyses.

b. Eggers p for possible publication bias 0.498 indicated low risk.

c. Influence analysis of the standardised mean results of these trials indicated that two studies (Sartore 2009 and Babiker 2018) were identified an influencing the pooled result. The pooled result without these two studies was MD -1.59 cm (95%CI -3.07 to -0.10).

d. Univariate meta regression analysis did not identify a difference in pooled results due to an any underlying difference between trials beyond a change in fibre intake.

**Authors**: Andrew N Reynolds, Ashley Akerman, Jim Mann

**Date**: 8 December 2019

**Question**: What is the effect of increasing dietary fibre intakes on fasting plasma insulin (standardised mean difference) in diabetes management?

**Setting**: Adults with prediabetes or type 2 diabetes participating in intervention trials, searches run January 2019.

S18 Table K

| **Certainty assessment** | | | | | | | | **№ of patients** | | **Effect** | **Certainty** |
| --- | --- | --- | --- | --- | --- | --- | --- | --- | --- | --- | --- |
| **Subgroup** | **№ of trials** | **Study design** | **Risk of bias** | **Inconsistency** | **Indirectness** | **Imprecision** | **Other** | **Intervention** | **Control** | **Standardised Mean Difference (95% CI)** |  |
| Overall | 19 | controlled trials | not serious ^a,b^ | serious ^c^ | not serious | not serious | none | 489 | 458 | 2.03 lower (2.92 lower to 1.13 lower) | ⨁⨁⨁ MODERATE |
| Mean difference in insulin (SMD) by global region | | | | | | | | | | | |
| Europe | 8 | controlled trials | not serious | serious ^d^ | not serious | serious ^e^ | none | 170 | 144 | 0.65 lower  (1.71 lower to 0.41 higher) | ⨁⨁ LOW |
| Asia | 6 | controlled trials | not serious | serious ^f^ | not serious | not serious | none | 217 | 218 | 2.14 lower  (3.48 lower to 0.80 lower) | ⨁⨁⨁ MODERATE |
| Middle East | 4 | controlled trials | not serious | serious ^g^ | not serious | serious ^e^ | none | 87 | 83 | 6.22 lower  (10.84 lower to 1.61 lower) | ⨁⨁ LOW |

**CI:** Confidence interval; **SMD:** Standardised mean difference.

#### Explanations

a. Eggers p for possible publication bias 0.011. Trim and fill analysis changed the observed SMD to -2.48 (95%CI -3.56 to -1.42).

b. Influence analysis of the standardised mean results of these trials identified one study (Lankinen 2001) that significantly influenced the pooled result. Removing Lankinen 2001 from the pooled result changed the SMD to -2.19 (-3.07 to -1.32).

c. Subgroups are shown in this GRADE table where a univariate meta regression analysis identified a difference in pooled results due to an underlying difference between trials.

d. Initial heterogeneity as measured by I^2^ was 93.9%.

e. The number of participants in this pooled estimate failed to meet the optimal information size necessary to avoid risk of imprecision.

f. Initial heterogeneity as measured by I^2^ was 96.4%.

g. Initial heterogeneity as measured by I^2^ was 98.0%.

**Author**: Andrew N Reynolds, Ashley Akerman, Jim Mann

**Date**: 8 December 2019

**Question**: What is the effect of increasing dietary fibre intakes on HOMA IR (mg/dL) in diabetes management?

**Setting**: Adults with prediabetes, type 1 diabetes, or type 2 diabetes participating in intervention trials, searches run January 2019.

S18 Table L

| **Certainty assessment** | | | | | | | | **№ of participants** | | **Effect** | **Certainty** |
| --- | --- | --- | --- | --- | --- | --- | --- | --- | --- | --- | --- |
| **Subgroup** | **№ of trials** | **Study design** | **Risk of bias** | **Inconsistency** | **Indirectness** | **Imprecision** | **Other** | **Intervention** | **Control** | **Mean difference (95% CI)** |  |
| Overall | 9 | controlled trials | not serious ^a,b^ | serious ^c^ | not serious | not serious | none ^d^ | 292 | 289 | 1.24 mg/dL lower (1.72 lower to 0.76 lower) | ⨁⨁⨁ MODERATE |

**CI:** Confidence interval; **MD:** Mean difference

#### Explanations

a. Eggers p for possible publication bias 0.219 indicated low risk.

b. Influence analysis of the standardised mean results of these trials identified one study (Lankinen 2001) that significantly influenced the pooled result. Removing Lankinen from the pooled result changed the MD to -1.41 (-1.91 to -0.92).

c. Univariate meta regression analysis did not identify a difference in pooled results due to an any underlying difference between trials beyond a change in fibre intake.

**Author**: Andrew N Reynolds, Ashley Akerman, Jim Mann

**Date**: 8 December 2019

**Question**: What is the effect of increasing dietary fibre intakes on systolic blood pressure (mmHg) in diabetes management?

**Setting**: Adults with prediabetes, type 1 diabetes, or type 2 diabetes participating in intervention trials, searches run January 2019.

S18 Table M

| **Certainty assessment** | | | | | | | | **№ of participants** | | **Effect** | **Certainty** |
| --- | --- | --- | --- | --- | --- | --- | --- | --- | --- | --- | --- |
| **Subgroup** | **№ of trials** | **Study design** | **Risk of bias** | **Inconsistency** | **Indirectness** | **Imprecision** | **Other** | **Intervention** | **Control** | **Mean difference (95% CI)** |  |
| Overall | 12 | controlled trials | not serious ^a,b^ | serious ^c^ | not serious | not serious | none | 325 | 295 | 1.86 mmHg lower (4.85 lower to 1.12 higher) | ⨁⨁⨁ MODERATE |

**CI:** Confidence interval; **MD:** Mean difference

#### Explanations

a. Eggers p for possible publication bias 0.972 indicated low risk.

b. Influence analysis of the standardised mean results of these trials identified one study (Babiker 2018) that influenced the pooled result. Without Babiker 2018 the pooled estimate was MD -1.5 mmHg (95%CI -4.6 to 1.7).

c. An initial univariate meta regression identified that our imputed correlation coefficients were too conservative, so we increased the imputed values by 10%.

**Author**: Andrew N Reynolds, Ashley Akerman, Jim Mann

**Date**: 8 December 2019

**Question**: What is the effect of increasing dietary fibre intakes on diastolic blood pressure (mmHg) in diabetes management?

**Setting**: Adults with prediabetes, type 1 diabetes, or type 2 diabetes participating in intervention trials, searches run January 2019.

S18 Table N

| **Certainty assessment** | | | | | | | | **№ of participants** | | **Effect** | **Certainty** |
| --- | --- | --- | --- | --- | --- | --- | --- | --- | --- | --- | --- |
| **Subgroup** | **№ of trials** | **Study design** | **Risk of bias** | **Inconsistency** | **Indirectness** | **Imprecision** | **Other** | **Intervention** | **Control** | **Mean difference (95% CI)** |  |
| Overall | 12 | controlled trials | not serious ^a,b^ | serious ^c^ | not serious | not serious | none | 325 | 295 | 1.19 mmHg lower (2.87 lower to 0.49 higher) | ⨁⨁⨁ MODERATE |

**CI:** Confidence interval; **MD:** Mean difference

#### Explanations

a. Eggers p for possible publication bias 0.891 indicated low risk.

b. Influence analysis of the standardised mean results of these trials identified one study (Babiker 2018) influenced the pooled result. Without Babiker 2018 the pooled estimate was MD -1.07 mmHg (95%CI -2.98 to 0.84).

c. Univariate meta regression analysis did not identify a difference in pooled results due to an any underlying difference between trials beyond a change in fibre intake.

**Author**: Andrew N Reynolds, Ashley Akerman, Jim Mann

**Date**: 31 May 2019

**Question**: What is the effect of increasing dietary fibre intakes on C-reactive protein (standardised mean difference) in diabetes management?

**Setting**: Adults with prediabetes, type 1 diabetes, or type 2 diabetes participating in intervention trials, searches run January 2019.

S18 Table O

| **Certainty assessment** | | | | | | | | **№ of participants** | | **Effect** | **Certainty** |
| --- | --- | --- | --- | --- | --- | --- | --- | --- | --- | --- | --- |
| **Subgroup** | **№ of trials** | **Study design** | **Risk of bias** | **Inconsistency** | **Indirectness** | **Imprecision** | **Other** | **Intervention** | **Control** | **Standardised mean difference (95% CI)** |  |
| Overall | 7 | controlled trials | not serious ^a,b^ | serious ^c^ | not serious | not serious | none ^d^ | 216 | 217 | 2.80 lower (4.52 lower to 1.09 lower) | ⨁⨁⨁ MODERATE |
| Mean difference in CRP when participation wasn’t restricted by HbA1c level | | | | | | | | | | | |
| Not restricted | 6 | controlled trials | not serious | serious ^e^ | not serious | not serious | none | 202 | 203 | 2.68 lower  (4.56 lower to 0.79 lower) | ⨁⨁⨁ MODERATE |

**CI:** Confidence interval; **MD:** Mean difference

#### Explanations

a. Eggers p for possible publication bias 0.081 indicated low risk.

b. Influence analysis of the standardised mean results of these trials identified one study (Grunberger 2007) influenced the pooled result. Without Grunberger 2007 the pooled estimate was SMD -3.63 (95%CI -4.59 to -2.66).

c. Subgroups are shown in this GRADE table where a univariate meta regression analysis identified a difference in pooled results due to an underlying difference between trials.

d. Insufficient data were available to consider a non linear restricted spline analysis.

e. Initial heterogeneity as measured by I^2^ was 97.3%.
