# Supplementary material for: Dietary fibre and whole grains in diabetes management: Systematic review and meta-analyses
Source: PLoS Med. 2020 Mar 6;17(3):e1003053. doi: 10.1371/journal.pmed.1003053 (PMC7059907; doi:10.1371/journal.pmed.1003053)
Supplement: Attachment — Submitted filename: reynolds.pdf [file pmed.1003053.s020.pdf]

This paper reports a systematic review with meta–analysis of the association between dietary fibre and various diabetes related parameters.

I was asked for a statistical report and I interpret that to include all aspects of the design and conduct of the study.

Points of detail

Page 4 'for the first time', perhaps 'first time to our knowledge'?

Page 6 I suspect variance is missing between inverse and models.

Page 7 Strictly speaking what the regression tests detect is small study effects, calling it publication bias assumes you know the aetiology.

Page 7 Perhaps cite the actual R package used?

Page 8 What policy was adopted for deciding which duplicate to use? From the supplement it looks as though the chronologically first publication was used. Is that true? If it is it seems mildly eccentric, perhaps say why?

Page 8 There are quite a few influence measures one could use (see Viechtbauer and Cheung (2010) for a list) so which exactly was chosen?

Page 9 Since there were only two prospective studies I was a bit puzzled by the number of data–points in Figure 2. Perhaps clarify what they are? Is this explained by the multi–country nature of EPIC?

Page 10 and Figure 4 I am a bit baffled here. Is this a meta–regression on prescribed fibre intake or is it a synthesis of multiple results from some studies which used multiple doses of fibre?

Page 14 I wonder whether it might be better instead of presenting results subset by the categorical predictor in the meta–regression to present the relative difference and its confidence interval.

Page 20 Nice to see the extensive list of translators mentioned.

References It might be a kindness to the reader to indicate which of these are not in English to save people trying to retrieve something they cannot read. Reference 59 seems to be the only one with the title in brackets. In reference 48 I was intrigued by yi zhi. Is this a typo?

Supplementary tables Just presenting the p–values means that we do not know the direction of effect. For instance in Table S3 we see that

region affected the relationship between fibre and HbA1c but not which regions were involved nor which one(s) was/were different. I appreciate that the current article is comprehensively described with copious detail but I think we do need coefficients here with confidence intervals.

Supplementary figures Figures S12 and S14 suffer from quite a bit of overlap between the grey squares. Is there any way to scale these squares while still making them proportional to importance?

Although the summary estimates usually have narrow confidence intervals excluding the null some of them seem quite small effects. I have no idea of the value of reducing some of the clinical variables but reducing body weight by half a kilo, on average, does not seem very impressive.

Points of more substance

Multiplicity

Several of the forest plots reveal that multiple effect sizes come from a single study. Figure 3 is an example. This means that estimates are correlated. Since the authors have used R they can analyse these with a multivariate meta–analysis. There are several R packages which provide this functionality, I use metafor but there are others.

Heterogeneity

I would usually suggest providing confidence intervals for I^2 to show how it is imprecisely estimated but in this case with values so close to 100% I think we can ignore that. One thing which I would suggest is the use of a prediction interval as suggested in Riley et al. (2011)

The authors have done their best to explain the heterogeneity using meta–regression. I wonder whether the issue is that the primary studies are very precise which leads to very high values of I^2 all other things being equal. Rücker et al. (2008) discusses this.

Summary

Requests for clarification and some suggestions for enhanced analysis.

Michael Dewey

References

R D Riley, J P T Higgins, and J J Deeks. Interpretation of random effects meta–analyses. *British Medical Journal*, 342:964–967, 2011.

G Rücker, G Schwarzer, J R Carpenter, and M Schumacher. Undue reliance on I^2 in assessing heterogeneity may mislead. *BMC Medical Research Methodology*, 8(79), 2008.

W Viechtbauer and M W-L Cheung. Outlier and influence diagnostics for meta–analysis. *Research Synthesis Methods*, 1:112–125, 2010.
