# Supplementary material for: Dietary fibre and whole grains in diabetes management: Systematic review and meta-analyses
Source: PLoS Med. 2020 Mar 6;17(3):e1003053. doi: 10.1371/journal.pmed.1003053 (PMC7059907; doi:10.1371/journal.pmed.1003053)
Supplement: Attachment — Submitted filename: PlosMedicine Reviewers comments.docx [file pmed.1003053.s021.docx]

**Reviewer #1:**

This manuscript presents results of a systematic review and meta-analysis of dietary fiber in diabetes management. The rationale for conducting the study is well-developed. The authors included 2 prospective studies and 41 intervention trials, and demonstrated the benefits of increasing dietary fiber for reducing mortality and a series of cardiometabolic risk factors in prediabetes, type 1 and 2 diabetes. The statistical analysis is adequate and the potential sources of bias are well considered for each outcome. Here are some major concerns about the study:

1.      The number of prospective studies may be too small for a meta-analysis. Therefore, the authors may want to focus on clinical trials (I found one previous publication in Plos Medicine might be informative. PMID: 27434027), and provide some comments two prospective studies, In addition, causal inferences (such as "Cardiovascular mortality risk reduction" in line 169) can rarely be made from observational studies, and more conservative description would be preferred. If the authors want to keep meta-analysis of prospective studies, then influence tests are strongly discouraged as there are only two studies.

Both the prospective cohort studies were run in multiple European countries which enabled us to use country specific data for the larger of the two prospective studies (EPIC). Authors of both the studies provided us with additional information. The text has been expanded in the methods section on lines 171, the results section lines 222 and 255, and the discussion lines 465-468.

In response to the referee’s comment regarding causal inference, we believe that our overall conclusions have been conservative. With regards to the findings concerning cardiovascular mortality risk, we have become even more cautious by specifically mentioning on lines 420-421 and lines 472 that the number of events was small and the upper confidence interval remained above 1.

We consider that the availability of data from multiple separate European countries justifies the use of sensitivity analyses such as influence analysis. The influence analysis identified no difference in the risk reduction trajectories between country and cohort estimates.

2.      Some believes that a meta-analysis should not be performed when between-study heterogeneities are large(as in this study), because it shows that findings from these studies are essentially different and cannot be solely explained by random errors. The authors may want to defense their decision to perform a meta.

This is a crucial consideration of this study, we thank the reviewer for raising it. Our research question (does increasing fibre intakes improve diabetes management) is necessarily broad in order to inform dietary guidelines. Thus heterogeneity was expected. It was for this reason that we undertook multiple sensitivity analyses, meta regression and dose response testing. Sensitivity testing identified that fibre dose and baseline fibre intakes were key determinants of many of the associations between fibre and health outcomes. There was no suggestion that systematic bias or error explained the reported associations. As we could not identify consistent reasons for the majority of heterogeneity, we downgraded the strength of evidence in the GRADE tables for each trial outcome. We have discussed this further in the manuscript (lines 185-192, 277-289, 322-328, 347-352, 358-363, 393-396) and in the discussion on lines 488-502.

3.      The generalization of conclusion to type 1 diabetes has been listed as a strength of while only two studies have been conduced among T1D patients. The authors may want to comment on if dietary advices for T1D and T2D management are interchangeable, and why an analysis stratified by diabetes type was not performed.

The analysis performed by diabetes type was a meta regression analysis within the pooled result, comparing data from trials of type 1 diabetes with data from trials of type 2 diabetes, prediabetes, and trials which included people with type 2 and prediabetes. We considered this to be a more stable and robust method than stratifying by diabetes type given the small number of trials with type 1 diabetes identified. The relationship between dietary fibre and glycaemic control or other cardiometabolic risk factors (other than insulin measures where we did not include trials of participants with type 1 diabetes) did not differ between people with type 1 and those with prediabetes or type 2 diabetes.

4.      Except for mortality and glycemic control, 11 additional cardiometabolic risk factors were discussed in this study. The results of dose response analysis for each outcome in the manuscript text were so brief that need to be further discussed.
Thank you for this, we have expanded the text (lines 286 and 345-346) but also note that these associations are shown in the supplementary material. For trials we were conservative as to what we described as a dose response relationship, instead preferring to show the curves themselves for readers to consider the nature of the relationship between fibre intake and change in outcome.

Minor concerns:
Line 75, might be useful to mention which CVD risk factors have been considered.

Thank you, we have expanded the text relating to this point accordingly on lines 111-112.

Line 76, "and whether these benefits translate into a reduction in morbidity and mortality" How has this question been addressed ? Does this imply the inclusion of prospective cohort studies ?

Yes that is what this sentence implies. We have clarified this on line 113.

Line 175, "This resulted in an absolute risk reduction of 14 (95%CI 4 to" how was this data derived ?

Absolute risk reductions were obtained following GRADE protocols (added line 216) and shown in full in the GRADE tables (Appendices S18).

Line 185, it might be useful to describe some study characteristics briefly, such as sample size, doses, % men/women, disease status(T1D/T2D), and duration of intervention.

Thank you for this suggestion, the text has been modified accordingly on lines 222-226.

The flow chart (Figure 1) is confusing-- out of 8,050 records retrieved from initial search, however, the sum of the records from different databases below is 8,086; 42 studies were included according to the flow chart while there are 43 identified studies in Table S1 and Table S2.
We are unsure how this has occurred, and have traced back the numbers to provide the correct information (total number 8086). Thank you for pointing this out. There are 42 studies listed in tables S1 and S2, however 43 references. This is because we recognised two papers from Babiker et al. (2017 and 2018) under the same listing as they report on the same data set. There is a footnote to table S2 explaining this.

**Reviewer #2:**

This paper reports a systematic review with meta–analysis of the association between dietary fibre and various diabetes related parameters. I was asked for a statistical report and I interpret that to include all aspects of the design and conduct of the study.

Page 4 ‘for the first time’, perhaps ‘first time to our knowledge’?

Thank you yes, the journal editor also commented on this point. We have adjusted accordingly on line 119.

Page 6 I suspect variance is missing between inverse and models.

Thank you, this has been corrected.

Page 7 Strictly speaking what the regression tests detect is small study effects, calling it publication bias assumes you know the aetiology.

While the term ‘publication bias’ is frequently used in this context in comparable publications we accept the reviewers expertise and have modified the sentence accordingly on line 211-212.

Page 7 Perhaps cite the actual R package used?

Thank you for highlighting this omission. We used the ‘metafor’ package for analyses in R. The text has been modified to include the R package used on line 214.

Page 8 What policy was adopted for deciding which duplicate to use? From the supplement it looks as though the chronologically first publication was used. Is that true? If it is it seems mildly eccentric, perhaps say why?

Duplicates in searches come about from combining results from multiple search engines identifying the same papers, and are automatically removed by the screening software used (Covidence) which matches title, author, year of publication, and journal. We have specified the use of this software on lines 157-158.

Page 8 There are quite a few influence measures one could use (see Viechtbauer and Cheung (2010) for a list) so which exactly was chosen?

One by one each individual effect size was omitted from an analysis to see if it shifted the pooled results. As such this sensitivity analysis identifies both influence and outlying results as discussed in Viechtbauer and Cheung 2010. This is now mentioned on lines 211-212. We considered that excessive influence may exist if the point estimate of an omitted analysis sits outside the confidence intervals of the pooled analysis. When this occurred, the pooled effect size with and without the influential study is reported in the footnotes of the GRADE table (Appendices S18). Where the removal of an influential study changes the direction or significance of a pooled result, the strength of the body of evidence is downgraded once for risk of bias following GRADE protocols.

Page 9 Since there were only two prospective studies I was a bit puzzled by the number of data–points in Figure 2. Perhaps clarify what they are? Is this explained by the multi–country nature of EPIC?

Thank you for this point, we have now stated in the manuscript (lines 171, 221, and 248) that both cohorts were from multiple countries in Europe, and we used the country-specific effect sizes from the EPIC cohort.

Page 10 and Figure 4 I am a bit baffled here. Is this a meta–regression on prescribed fibre intake or is it a synthesis of multiple results from some studies which used multiple doses of fibre?

Figure 3 on page 13 summarises trials reporting the mean difference between intervention and control. Some included several relevant intervention arms which are shown in this figure. We have discussed the issue of multiplicity in more detail in response to your comment below.

Figure 4 on page 15 presents dose response relationships based on meta regression analyses with restricted cubic splines. The data for HbA1c and fasting plasma glucose are shown in relation to the ‘doses’ representing fibre intakes expressed as a percentage of baseline fibre intake. We did this because univariate meta regression testing identified the baseline fibre intake as relevant to the pooled results. This has now been specified in a footnote to the figure.

Page 14 I wonder whether it might be better instead of presenting results subset by the categorical predictor in the meta–regression to present the relative difference and its confidence interval.

We acknowledge this suggestion. However we believe that presenting data for the relevant subsets and the number of trials for each subset is slightly more informative, and potentially less likely to be confused. The findings from the meta regression analyses have been moved up in the results section and expanded to better report the results for each outcome.

Page 20 Nice to see the extensive list of translators mentioned.

Thank you! We could not have considered these trials without them.

References It might be a kindness to the reader to indicate which of these are not in English to save people trying to retrieve something they cannot read. Reference 59 seems to be the only one with the title in brackets.

Excellent idea, we have modified in the references of the manuscript as well as in the table of identified studies in the supplementary material.

In reference 48 I was intrigued by yi zhi. Is this a typo?

This wording was included in the reference in one of the three repositories we found that held the reference, but not the other two. It has now been removed, thank you for pointing this out (line 756).

Supplementary tables Just presenting the p–values means that we do not know the direction of effect. For instance in Table S3 we see that Page 1 Reynolds et al August 21, 2019 region affected the relationship between fibre and HbA1c but not which regions were involved nor which one(s) was/were different. I appreciate that the current article is comprehensively described with copious detail but I think we do need coefficients here with confidence intervals.

Thank you for this observation, it touches upon something you mentioned earlier. The subgroup estimates and confidence intervals where a meta regression identified a difference from the overall results are shown in the GRADE tables (appendices S18). We have tried to show as much as possible that would be informative to the reader, but not unnecessarily repeat data. As these subgroups must be shown in the GRADE tables to inform dietary guideline development, we have not shown them again within other parts of the supplementary material.

Supplementary figures Figures S12 and S14 suffer from quite a bit of overlap between the grey squares. Is there any way to scale these squares while still making them proportional to importance?

Absolutely, changed now, sorry this was not done initially. We have changed the look of all forest plots within the supplementary information so that they are uniform. Included trials and effect sizes have not changed from the initial submission.

Although the summary estimates usually have narrow confidence intervals excluding the null some of them seem quite small effects. I have no idea of the value of reducing some of the clinical variables but reducing body weight by half a kilo, on average, does not seem very impressive.

Given that this is an average result of many people, this finding would be considered to be clinically meaningful. A small reduction in body weight can be associated with appreciable improvements in glycaemic control.

**Points of more substance**

Multiplicity Several of the forest plots reveal that multiple effect sizes come from a single study. Figure 3 is an example. This means that estimates are correlated. Since the authors have used R they can analyse these with a multivariate meta–analysis. There are several R packages which provide this functionality, I use metafor but there are others.

Regarding statistical packages: both Stata and R (metafor) were used as stated, however the primary program used was Stata. R was used to double check much of our initial results and sensitivity analyses, and to generate some figures.

Regarding multiplicity: we followed Cochrane recommendations. We first ran initial pooled analyses where all estimates from a paper were first combined with fixed effects models and then combined with other studies with a random effects model (a single pair-wise comparison). The results of doing so did not change the direction or significance of the results that we have reported. We show pooled estimates which include the results of each relevant intervention identified in the manuscript. To do this, we split the size of the ‘shared’ control group, which led to a greater variance around the mean difference. This is a recommended method to avoid a unit of analysis error (handbook 16.5.4). We used each of the intervention results in our meta regression analyses, and having accounted for the potential for multiplicity, chose to show the results of each intervention as well. These steps are now reflected in the manuscript, lines 179-182.

Heterogeneity I would usually suggest providing confidence intervals for I 2 to show how it is imprecisely estimated but in this case with values so close to 100% I think we can ignore that. One thing which I would suggest is the use of a prediction interval as suggested in Riley et al. (2011) The authors have done their best to explain the heterogeneity using meta– regression. I wonder whether the issue is that the primary studies are very precise which leads to very high values of I 2 all other things being equal. R¨ucker et al. (2008) discusses this.

Thank you for these insightful comments, we were very interested to read the papers by Rücker et al. and Riley et al. Rücker er al. 2008 considers statistical heterogeneity, however we believe what we are seeing is clinical baseline heterogeneity and heterogeneity from other sources as a result of our broad research question. Our research question was necessarily broad in order to inform clinical guidelines for diabetes management, as such it identified trials that have used different fibres at different doses on participants with different types of diabetes and on different medications. Due to this diversity we did not expect a consistent underlying effect size, hence our decision to use a random effects model. We have shown the average treatment effect and its confidence intervals, rather than prediction intervals (Riley et al. 2011) for the same reason.

Summary Requests for clarification and some suggestions for enhanced analysis.

References R D Riley, J P T Higgins, and J J Deeks. Interpretation of random effects meta–analyses. British Medical Journal, 342:964–967, 2011.

G R¨ucker, G Schwarzer, J R Carpenter, and M Schumacher. Undue reliance on I 2 in assessing heterogeneity may mislead. BMC Medical Research Methodology, 8(79), 2008.

W Viechtbauer and M W-L Cheung. Outlier and influence diagnostics for meta–analysis. Research Synthesis Methods, 1:112–125, 2010.

**Reviewer #3:**

The systematic review and meta-analysis from Reynolds et al titled: "Dietary fibre in diabetes management: systematic review and meta analysis" provides relevant and comprehensive update on previous literature in this area on a wide scope of outcomes.   However, there are several concerns that need to be addressed, outlined below:

-       In the introduction, the authors rationalize the need for the systematic review and meta analysis through dietary CHO focus, citing that (lines 63-65) controlled trials confirm potential of relatively high to low CHO intakes in diabetes. The latest SRMA however, does not fully support this stance.  Moreover, the SRMA cited from 2013 refer mostly to the benefit of fiber addition/supplementation rather than as part of a high CHO diet.  The authors should provide some rationale for the trial in the context of more recent research/meta analysis on whole grain/dietary fibre in DM management (McRae MP. J Chirop Med. 2018; Wang Y et al. Int J Mol Epid Genet. 2019; Jovanovski E et al, Diabetes Care, 2019,e tc ).

The introduction recognises the historical perspective of carbohydrate, both in terms of amount and quality. It then narrows to discuss the context of the current review which relates to quality (increasing dietary fibre) without purposeful manipulation of quantity. This topic has not been addressed since 2013. The more recent reviews, some of which are now mentioned in the introduction on lines 102-104, and the discussion lines 436-438, address very separate research questions. Wang et al. 2019 considers wholegrain and cereal fibre intake and risk of type 2 diabetes, as have we previously (Reynolds et al. 2019). This is not diabetes management. Jovanovski 2019 compared viscous fibres supplementation with no fibre or insoluble fibre in diabetes management, a worthy question but ultimately separate from our review in terms of research question, intervention type, populations included, and outcomes measured. McRae et al. 2018 conducted an umbrella review that considered both type 2 diabetes incidence and blood glucose control in diabetes management, but as an umbrella review did not update meta analyses in this area as we have done.

Given the focus of our introduction is the broader context in which carbohydrates are consumed, we then come back to consider quality and quantity in the discussion.

-       With regards to analysis of mean difference (MD) between intervention and control groups (line 131), please indicate whether MD between end values or MD between change-from-baseline values were used and whether there was an order of preference. I.e. order of preference could be use of change values provided by article if available, followed by calculation of change values where appropriate, followed by end values provided by article and lastly calculation of end values.

MD between change-from-baseline values were used first, followed by calculation of change values where appropriate. This has been added as suggested on line 179-180.

-       Please provide detail on how the trials with multiple comparison arms were treated? If compared to a single control arm, were the comparisons combined to create a single pair-wise comparison or were they included as separate comparisons? If the latter, were there any adjustments made (ie. was the SE(MD) adjusted) to take this duplicate comparison into account? I.e. In the forest plot for HbA1c (figure 3) it appears that multiple intervention arms from the same trial were listed separately.

In the instances where this occurred, the multiple interventions either differed in nature (3 trials), or fibre amount (2 trials), or participant groups were reported separately by a genetic factor or by medical treatment (3 trials). We followed Cochrane guidelines in order to avoid a unit of analysis error (Cochrane Handbook 16.5.4). We ran initial pooled analysis where all estimates from a paper were first combined with fixed effects models and then combined with other studies with a random effects model (a single pair-wise comparison). The results of doing so did not change the direction or significance of the results that we have reported.

What we show in the manuscript are the estimates from all eligible interventions, where the ‘shared’ control group has been split in size leading greater variance around the mean difference. We have added more details to the methodology lines 180-182 to address this comment.

-       In line 144, please elaborate on the influence analysis, i.e. what determined whether a study was influential?

This analysis identifies where a point estimate from an individual trial was outside the pooled confidence intervals in a model considering study weight (i.e point estimates that are both influence and outliers). When this occurred, we report the pooled results with and without the that trial in the GRADE tables (appendices S18). The Stata command for this test is metaninf.

-       Concern that the search is not comprehensive. The authors have used only "dietary fiber/re" or whole grain terms to try to 'comprehensively' capture all research on dietary fiber. It is likely that many possibly eligible trials were missed by not including explicit food search terms or specific known fibers/whole grains or their sources (ie for beans (legumes), oats, cereal etc)... It appears that trials fitting selection criteria have been missed (ie McGeoch et al., 2013 etc). The review here included many cardiometabolic outcomes, such as blood lipids, blood pressure, etc. However, the presented online search term only included glycemia-related search for outcomes (Search#2 Supplementary, pg 3).  Again, it is possible that some trials relating to these outcomes may have been missed.

Our search included the terms ‘Dietary carbohydrate OR whole grain OR wholegrain* OR whole-grain OR dietary fibre OR dietary fiber OR “ CHO ”. These terms were used as MeSH descriptors wherever possible. Our inclusions of wholegrain interventions is discussed in the manuscript and was done on the understanding that they are a major food source of dietary fibre. Food sources other than whole grains were not included as search terms.

McGeoch et al. 2013 was identified in the searches for this review but was not considered eligible for inclusion as dietary fibre was not increased.

Although the online search did not include all secondary outcomes, trials in which they were reported would have been detected through the strategy we used. Our use of handsearching of the reference lists of the relevant systematic reviews, prospective studies, and trials enabled us to consider all previously identified trials as well as identify additional reports of secondary outcomes (i.e. Dehghan et al. 2014, Babiker 2018) or publications that did not report directly on glycaemia (i.e. Pick et al. 1996).

-       Within the dose response analysis, how did the authors collectively assess doses of whole grain sources ie 50g of barley, containing ~8% b-glucan fiber, relative to more isolated sources of dietary fiber?  Did the authors extrapolate/estimate the amount of fiber from whole food sources?   The dose response analysis would otherwise not have much merit.  In S1 under a priori subgroup analysis: Amount: is the volume of fibre or whole grain used?

All dose response testing involved the amount of fibre and not the amount of whole grains. We relied on the published data or information obtained directly from the authors. This is stated on line 193.

-       A priori subgroup analysis included "fibre type: all fibre or just one type (i.e inulin) viscosity, solubility". Please clarify in more detail what fibre types were considered, which 'viscosity' and 'solubility. Is this an inclusive list of all the subanalysis that were performed?

We acknowledge that terms such as soluble or non-soluble are somewhat arbitrary and do not accurately represent where in the spectrum of solubility a fibre may sit. However we believed that it would be of interest to present this breakdown of dietary fibre given the widespread use of these terms in the current literature (e.g Jovanovski et al. 2019). We relied on the published data to identify what fibres were used.

-       In the GRADE assessment authors report on categorical analysis of study participant health status (ie presence/absence of renal issues etc). This was not included in the PICO table of a priori subgroup analysis?  Please indicate exactly which variables were tested, and whether these variables were determined apriori.

This is a fair point as the PICO table lists the a priori consideration of participant groups based on weight, age, sex, SES, medication type, as well as by diabetes type. However when reviewing the trials we found that they varied as to whether or not comorbidities were excluded. Because this potentially has an important impact on the generalisability of our results, we considered it was important to assess. We have modified the methods section to clarify the inclusion of this meta regression on lines 185-192.

-       In table S3 authors exclude based on HbA1c, aged over 65 etc. Is this categorically assessed (ie over vs uder 65?). What does exclusion based on HbA1c or BMI mean? Please be more concise in provision of explanations.

We have modified the text accordingly on page 19 of the supplementary material.

-       What singular fibre types were assessed as a dicotomous variables (is Table S1)?

Table S1 is a description of the two identified multi-country prospective observational studies.

Meta regression of trial data considered: fibre as a supplement/incorporated into food (dichotomous), fibre singular extract/ mixed (dichotomous), fibre viscous/non- viscous (dichotomous), and fibre soluble/non- soluble (dichotomous).

-       Were there any restrictions on study duration? It is questionable whether short term trials (ie <3-4wks) are relevant on certain outcomes, especially on your primary outcome.

The stated eligibility criteria required trials to be of at least six weeks duration (line 139). This was a decision approved by the DNSG of the EASD guideline group as it is the minimum duration of a trial where HbA1c (the primary outcome stated in the PICO table) might be expected to change. We have now stated this in the manuscript lines 140-142.

-       The dose response curve for HbA1c is presented as % intake from baseline. How much data was available?  The authors do not report on the dose response relationship per amount of fibre intake per se. Does the relationship still hold?

As stated in the methodology on line 194, “When we identified an interaction with baseline fibre intake we tested the fibre increase as a percentage of the baseline fibre intake”. This was the case for HbA1c and fasting plasma glucose. The curve for Hba1c was generated with data from 758 participants of 16 trials. We have updated the dose response curves of the manuscript (page 15) and throughout the supplementary information so that the number of participants and trials used to generate the curve is shown each time.

-       We need to be very careful in conclusions that the study results cannot be generalized to whole grain as only very few studies evaluated a whole grain.  Perhaps that identifies future areas of research.  Most of the data is on dietary fiber supplements and concentrates and this perhaps should be acknowledged.

Meta regression analysis used data from all trials and did not identify differences in outcomes between wholegrain studies and those of fibre supplements. Whole grains themselves are variable in the nutrients provided by grain type. We have further discussed the use of such meta regression on (lines 185-192, 277-289, 322-328, 347-352, 358-363, 393-396) and in the discussion on lines 488-502.

-       Please add a section on study limitations. Some limitations are already mentioned in discussion.

We have substantially revised the section on limitations in response to this comment and to take into account the comments of the other reviewers. Lines 461-508.

-       Discussion: Lines 283-284. Was this statement based on subanalysis of background treatment type (ie diet alone vs oral agents, vs insulin vs combination) for each of the 5 outcomes?

Yes, these analyses are available for each outcome in the supplementary material. Subgroup results identified as significant are shown in the GRADE tables appendices S18.

-       Discussion: Line 324-325. Please provide data on MD according to each category (T1DM, T2DM, pre-diabetes). There are very few studies in the non-T2DM categories.

Meta regression failed to identify a difference in effect size for outcomes due to diabetes type. We therefore consider it inappropriate to present MDs for the different types of diabetes.

-       Discussion: Line 346:  The authors mention previous meta analysis of RCTs demonstrating  potential of psyllium and viscous fibers to affect glycemic control. These SRMAs report on significantly larger magnitudes of change (ie the latter SRMA, the effect of viscous fiber is approx. 3 fold higher, including both extracted fibers and whole foods -ie oats).  It would be useful to comment on the difference observed and potential of different fiber types to improve magnitude of effect of fiber, rather than focus on all fiber that is highly heterogeneous (as demonstrated here).

The research question and eligibility criteria are quite different between reviews, making it difficult to draw useful comparisons between the two. We reference Jovanovski et al. 2019 in our introduction and discussion as a very useful consideration of the effects of viscous fibre (when compared to no fibre or insoluble fibres) on Hba1c and FPG in adults with type 2 diabetes. As stated by the reviewer their effect size was greater, an unsurprising findings as their research question was very different. In conducting our review we found some crossover with the eligibility criteria of Jovanovski et al. 2019, however there were differences as well. Trials of viscous fibre supplements compared with no increase in fibre that may have been relevant to both reviews, but were only included in our review were Calvo-Rubio et al. 1987, Sartore 2009, and Stahl 1990 (the guar arm). Schwab 2006 also has a pectin intervention where data for only those participants with type 2 diabetes may have been obtained on request from the authors. It is not clear why these studies were not mentioned in the review by Jovanovski et al. Each of these trials produced little to no effect on HbA1c, had they been deemed eligible for inclusion in the Jovanovski et al. 2019 review, the pooled effect size would have been appreciably reduced. Heterogeneity for the two measures compared by both reviews were >90% when measured by I^2^.

-       Lines 356-360. Similarly, we need to be careful when extrapolating conclusions on cardiometabolic risk (as the 'only direct evidence for such benefit") based on 2 cohorts that have inherent risk of confounding and in this instance, have not shown a cardiovascular benefit. While the authors rightly conclude that the consumption of high fibre foods should be encouraged (line 361), the conclusions should be in the context of the current evidence from RCTs and fiber sources they studied.

Both cohorts were multi country, for the EPIC cohort which was quite large, we considered the per country estimates. This issue has been discussed in further detail to Reviewer 1.

-       The authors comment in the discussion on reduced risk of total mortality, but not on results of CV mortality which is the focus of the assessed RF in this review.

Results for both all-cause and CVD mortality from prospective observational studies are shown in Table 2 of our manuscript. Although the dose response curves for these two outcomes are very similar (P wald test 0.811), the upper confidence interval of the association between fibre and cardiovascular mortality remained above 1 due to the smaller number of events. It was for this reason that we did not wish to overemphasise this finding in the discussion, however due to your comment we have now added this to this discussion on lines 420-421 and 470-474.

-       Discussion: Lines 293-304 and 363-372 further discuss comparison of low/high carbohydrate diets which are not considered or explored in this SRMA (as sub-analysis of background diet) and thus may not be very relevant in this context. The fiber in majority of trials is supplemented to the diet. These sections should be less elaborate.

Given the current interest in carbohydrates for both amount and quality, we believe these sentences provide necessary context to the work we have done.

-       Lines 305-305: The authors suggest that the weight-loss may be 'principally responsible' for the observed effect on HbA1c. However, the MD in weight-loss reported here and in other SRMAs of dietary fibre is minimal (MD of <1kg) and likely not sufficient to generate the HbA1c reduction observed in this and other SRMAs.

A very modest weight loss can appreciably influence glycaemic control. This has been discussed in response to a comment by Reviewer 2.

-       Minor: Figure 3 describing mean difference in HbA1c contains random descriptors of study (ie either dose, type of fiber, diet in brackets or no descriptor, without apparent systematical order. Please revise.

Thank you we have revised this figure.

-       Minor: Line 138: Prescribed or measured fibre increase was used in dose response testing. Which was taken when both are reported?

We considered prescribed and measured separately, however our priority was measured given compliance with what is prescribed will not be 100%. We have clarified this on line 193.

**Requests from the editors:**
Abstract please combine methods and findings into one section

This has been done line 26.
Abstract-last sentence of the methods and findings section should include the limitations of your methodology

This has been done line 56-58.
Abstract- last sentence “… reduce risk of premature mortality”. Should this be followed by…” in diabetic patients?”

Thank you, this has been done line 65.
At this stage, we ask that you include a short, non-technical Author Summary of your research to make findings accessible to a wide audience that includes both scientists and non-scientists. The Author Summary should immediately follow the Abstract in your revised manuscript. This text is subject to editorial change and should be distinct from the scientific abstract.

This has been added lines 67-86.
Please include an “Introduction” heading at the start of the introduction

This has been done line 88.
Line 70- please remove reference to “new” or clarify if this is an update to a previously published systematic review. Please provide a citation for the same.

The word new has been removed line 105.
Line 72- “has” not “have”

Good point, this has been changed.
Line 77-78 please avoid assertions of primacy

This has been changed line 107.
Page 7- please move the role of the funding source to the “Financial disclosure” section

This has been moved to the financial disclosure section of the online portal.
Please provide p values along with 95% confidence intervals where appropriate

We believe where we have reported P values when it is appropriate to do so.

Please present and organize the Discussion as follows: a short, clear summary of the article's findings; what the study adds to existing research and where and why the results may differ from previous research; strengths and limitations of the study; implications and next steps for research, clinical practice, and/or public policy; one-paragraph conclusion.

This has been done.
Line 277- a systematic review cannot demonstrate any health benefit, please revise as necessary

This has been corrected line 414.
Line 280-281-please clarify if the reduced risk of mortality is for the general population or for patients with diabetes

This has been clarified line 416.
Please remove page numbers from the PRISMA checklist as these are likely to change
These have been updated (last page of supplementary material).
