# Supplementary material for: Dietary fibre and whole grains in diabetes management: Systematic review and meta-analyses
Source: PLoS Med. 2020 Mar 6;17(3):e1003053. doi: 10.1371/journal.pmed.1003053 (PMC7059907; doi:10.1371/journal.pmed.1003053)
Supplement: Attachment — Submitted filename: PlosMed response to reviewers.docx [file pmed.1003053.s022.docx]

We expect to receive your revised manuscript by Dec 17 2019 11:59PM. Please email us ([plosmedicine@plos.org](mailto:plosmedicine@plos.org)) if you have any questions or concerns.

**Reviewer #2:** The authors have addressed my points.

I still feel that prediction intervals are helpful in the presence of heterogeneity because they tell us about what might be the result from a future study. By contrast the confidence intervals tell us about the precision of the overall mean. If on reflection the authors still feel they are not informative I would not want to push the issue.

We acknowledge the reviewer’s expertise in this area and will consider this suggestion in future research.

**Reviewer #3:** The authors have addressed some of the raised issues in sufficient manner. However, there are several concerns that remain which are very relevant and should be carefully considered before proceeding:

A concern remains that the search is not systematic and comprehensive. The authors state that food sources other than whole grain were not included as search terms. This limits the review to comprehensively searching only whole grain types of fiber. The authors should make this more prominent in their conclusions.  The reader should not be misguided to infer all dietary fiber was included.

The reviewer has not fully appreciated the purpose of our research which was to examine the effect of total dietary fibre. This objective is stated on **lines 116-119**.

The additional focus on whole grains (specified by the DNSG of the EASD) is because increasing wholegrain intakes is one of the most consistent messages in nutritional recommendation for people with diabetes. This focus did not diminish our capacity to identify eligible trials of dietary fibre regardless of source. Extending our search terms to include wholegrain consumption enabled us to test the validity of this message. See **lines 78-79** and the PICO table in **Appendix S1**.

Similarly, there are a number of cardiometabolic outcomes reported from trials in addition to glycemic measures. The search terms do not include these. While the authors suggest hand search was employed and they believe they would detect most through the glycemic search terms, this is not a systematic review. This is an important limitation.

We have augmented our initial searches in order to establish whether relevant publications had been missed. This new search was conducted in the same databases specified in the manuscript from database inception to January 18^th^, 2019 and included terms for all the cardiometabolic outcomes considered in this review. This step identified an additional 3,610 potential publications, of which 575 were duplicates. After screening by two reviewers independently as per what is stated in the manuscript, only 18 of these potential publications were considered in full text review. Two trials not previously identified were found to be eligible for the current review. Details of this search process have been added to the manuscript and supplementary material, with the two publications included in analyses, sensitivity analyses, and meta regression analyses when appropriate to do so. The additional data did not change the significance or direction of any results presented in the manuscript.

The last search was done in Jan 2019.  This approaches one year (from publication time). A search update is strongly recommended.

Following a discussion with the editorial team, this step was not undertaken.

The authors state that all dose response testing used the fiber dose.  In many trials, the fiber dose is not reported (ie when oats/whole grain is administered). If not available (ie directly from authors), how was the fiber dose estimated (ie of b-glucan in whole oats or barley, psyllium fiber)?

Where the amount of fibre was not provided in the published paper or obtained from the authors or manufacturers of the fibre, either in the amount measured (preferred) or the amount prescribed, these trials were not included in dose response testing. We have modified the sentence on **lines 176-178** to reflect that these data had to be available for the trial to be included in dose response testing.

A priori subgroup analysis included type of fiber, viscosity and solubility. The authors did not report on which fibers were categorized in these groups in order to run sub-analysis. Please clarify.

As mentioned when we initially responded to this comment from the reviewer, we acknowledge that terms such as soluble or insoluble are somewhat arbitrary and do not accurately represent where in the spectrum of solubility a fibre may sit. However we believed that it would be of interest to consider this breakdown of dietary fibre given the widespread use of these terms in the current literature (e.g Jovanovski et al. 2019). We have had no option other than to rely on the published data.

Following the upgrade of results with the two trials identified in the additional searches, the HDL results relating to viscosity were updated. Please see **lines 274-277**.

The overall tone and discussion of paper puts emphasis on whole grain. However, majority of trials appear to be supplementation of fiber from various sources to diet (and 2 cohorts).  While the authors argue that meta regression did not identify differences between 2 sources (p=0.057), we must be careful how we interpret the available evidence. Evidence, based on the present search for whole grain, is still scarce (only n=4 trials for HbA1c), and most of the data refers to supplemented type. The conclusions therefore need to be cautionary.

As explained in response to the first of this reviewer’s comments, our review relates to total dietary fibre (stated on lines **24, 27, 67, 101, 116**, and **121-122)**, with an additional consideration of the data on whole grains in order to increase the translation of our findings into practice. As well as confirming the standard dietary advice to replace refined grains with whole grains (**lines 78-79**), our conclusion states “those with all types of diabetes should be encouraged to have adequate intakes of dietary fibre. Vegetables, pulses, whole fruits, and whole grains are excellent sources.” (**line 450-453**). We do not consider that this is an over emphasis of whole grains as a source of dietary fibre.

Please note the P value that has been given is for a meta regression for fibre incorporated into food (P0.057 before results update) and not whether the fibre was provided from a wholegrain trial or not (P0.177 results update), and only for the outcome HbA1c. Our interpretation of findings relied on the results from all fourteen outcomes from the trials, as well as the findings from the prospective observational studies. Regarding a potential difference in the results between fibre provided from whole grains or from other sources, the quantitative test results were not significant for any of the fourteen outcomes from trials, with P values of the meta regressions ranging from 0.079 to 0.911 (following update).

Similarly, the concern remains that strong recommendations and conclusions in discussion are based on only 2 European cohorts (ie "the only direct evidence for benefit derives from fibre as it occurs naturally in food), whereas most of the evidence from controlled trials on cardiometabolic risk is on fibre supplement (if discussing natural fiber from foods, ideally all food sources including legumes should have been included in this review).

Direct evidence is measured in hard outcomes, such as premature mortality. Cardiometabolic risk factors are mediating factors in risk of a hard outcome occurring. The language used in the discussion is to reflect these differences.

As we have mentioned above the primary purpose of our work was to consider the effects of total dietary fibre. We can confirm that legume/pulse intake was included in the food frequency questionnaires of the cohort studies, and in the intervention arm of 6 of the 42 dietary fibre trials.

Despite that meta regression did not identify differences in effect size based on diabetes type, etc. for transparency purposes, information on MD + CIs for each subgroup estimate is warranted, especially in light of multiple comments in this regard.

This was discussed in some detail in our response to the statistical reviewer’s initial comments. In some detail, the analysis performed by diabetes type was a meta regression analysis within the pooled result, comparing data from trials of type 1 diabetes with data from trials of type 2 diabetes, prediabetes, and trials which included people with type 2 and prediabetes. We considered this to be a more stable and robust method than stratifying by diabetes type given the small number of trials with type 1 diabetes identified. Presenting stratified data where there is insufficient evidence to suggest a difference has the potential to be highly misleading.

The authors continue to suggest that the weight-loss observed here may be 'principally responsible' for the observed effect on HbA1c. The MD in body weight was -0.6kg. Guidelines generally cite that sustained weight loss of ≥5kg may improve glycemic control. If deciding to keep, please provide references for the statement in the response referring to <1kg change in weight.

This is an interesting interpretation of a component of our discussion, that has caused us to look at our phrasing and sentence structure. Because we identified a significant interaction when we tested the influence of weight control vs non-weight control studies on the overall HbA1c reduction, we cannot discount that this is a mediating factor in the relationship. This interaction was seen with the available data and occurred irrespective of what guidelines generally cite. Nevertheless we have modified this wording from “principally responsible” to “may have been” (**line 374**).

Please note in discussing this interaction, we have also provided an alternative explanation which is random chance due to the small number (five) of trials that reported on HbA1c and controlled for body weight. This cautious interpretation of the results appears appropriate, and in fact augments why we do not wish to show subgroups of data such as by diabetes type as requested in the comment above, especially when a significant interaction was not observed by diabetes type for any of the 14 outcomes considered.

The authors recommend that in those with prediabetes, type 1, or type 2 diabetes - should increase their dietary fibre intakes by 15 grams per day or to 35 grams per day.  Does this hold for each diabetes category? What is the basis for 15g?  The recommendations should be more cautionary (very little data for type1 DM exists).

The 15 grams per day increase comes from what was measured (**lines 159-161**), shown (**lines 213-216**), and then discussed (**lines 340-343**) in the current manuscript. We are unsure how to signpost this point further.

We tested for an interaction between diabetes type and the results for each of the 14 cardiometabolic risk factors included in this review with a categorical meta regression, with P values ranging from 0.130 to 0.961 (none significant). This quantitative consideration for each outcome supports the recommendation to be made for those with prediabetes, type 1, or type 2 diabetes.

Minor note: Sartore 2009 is not randomized trial. Please modify the Table of Characteristics.

This is a good point, thank you we have adjusted.

Minor note: It would be useful to provide the data for HbA1c in % (NGSP), perhaps in brackets for ease of reader.

The journal encourages the use of SI units, however we will happily do so should the journal considers this useful.

**Requests from the editors:**

Title- in accordance with comments from Ref (3)- please amend title to “Whole grain fibre and management of diabetes: a systematic review and meta-analysis”

We have explained in our responses above that we believe that this reviewer has not fully appreciated that our review refers to total dietary fibre. Our preference would be to retain the original title. However if the editor would prefer an alternative title we suggest: “Dietary fibre and whole grains in diabetes management: systematic review and meta analyses”.

Please include a space between text and reference brackets

Done.

Page 6-please avoid assertions of primacy

In response to the initial comment of reviewer we modified our statement “for the first time” to “for the first time to our knowledge” (**line 106**). We hope that the editors will agree that this avoids any assertion of primacy. We have also gone through and reduced the strength of wording in the discussion (primarily the conclusion) in line with this sentiment. We have done so largely by acknowledging that our findings support current nutritional guidelines.

Discussion- a systematic review cannot demonstrate any health benefit, please revise as necessary. Please also tone down conclusions to avoid overreaching.

In the first round of revisions we modified this sentence on your instructions from “Our systematic review and meta analyses have demonstrated” to “The findings from our systematic review and meta analyses have demonstrated”. This change provided accuracy to the statement.

Please update your literature search, in accordance with comments from Reviewer 3

Done, please see comment to reviewer above for further details.
